# The development of a fully-integrated immune response model (FIRM) simulator of the immune response through integration of multiple subset models

**DOI:** 10.1186/1752-0509-7-95

**Published:** 2013-09-28

**Authors:** Sirus Palsson, Timothy P Hickling, Erica L Bradshaw-Pierce, Michael Zager, Karin Jooss, Peter J O’Brien, Mary E Spilker, Bernhard O Palsson, Paolo Vicini

**Affiliations:** 1 GT Life Sciences, San Diego, CA, USA; 2 Department of Pharmacokinetics, Dynamics and Metabolism, Pfizer Worldwide Research and Development, Sandwich, UK; 3 Department of Pharmacokinetics, Dynamics and Metabolism, Pfizer Worldwide Research and Development, San Diego, CA, USA; 4 Current address: Department of Pharmaceutical Sciences, Skaggs School of Pharmacy, University of Colorado Anschutz Medical Campus, Aurora, CO, USA; 5 Vaccines Research Unit, Pfizer Worldwide Research and Development, San Diego, CA, USA

**Keywords:** Biological networks, Immune response, Mathematical modeling, Ordinary differential equation systems, Systems biology

## Abstract

**Background:**

The complexity and multiscale nature of the mammalian immune response provides an excellent test bed for the potential of mathematical modeling and simulation to facilitate mechanistic understanding. Historically, mathematical models of the immune response focused on subsets of the immune system and/or specific aspects of the response. Mathematical models have been developed for the humoral side of the immune response, or for the cellular side, or for cytokine kinetics, but rarely have they been proposed to encompass the overall system complexity. We propose here a framework for integration of subset models, based on a system biology approach.

**Results:**

A dynamic simulator, the Fully-integrated Immune Response Model (FIRM), was built in a stepwise fashion by integrating published subset models and adding novel features. The approach used to build the model includes the formulation of the network of interacting species and the subsequent introduction of rate laws to describe each biological process. The resulting model represents a multi-organ structure, comprised of the target organ where the immune response takes place, circulating blood, lymphoid T, and lymphoid B tissue. The cell types accounted for include macrophages, a few T-cell lineages (cytotoxic, regulatory, helper 1, and helper 2), and B-cell activation to plasma cells. Four different cytokines were accounted for: IFN-γ, IL-4, IL-10 and IL-12. In addition, generic inflammatory signals are used to represent the kinetics of IL-1, IL-2, and TGF-β. Cell recruitment, differentiation, replication, apoptosis and migration are described as appropriate for the different cell types. The model is a hybrid structure containing information from several mammalian species. The structure of the network was built to be physiologically and biochemically consistent. Rate laws for all the cellular fate processes, growth factor production rates and half-lives, together with antibody production rates and half-lives, are provided. The results demonstrate how this framework can be used to integrate mathematical models of the immune response from several published sources and describe qualitative predictions of global immune system response arising from the integrated, hybrid model. In addition, we show how the model can be expanded to include novel biological findings. Case studies were carried out to simulate TB infection, tumor rejection, response to a blood borne pathogen and the consequences of accounting for regulatory T-cells.

**Conclusions:**

The final result of this work is a postulated and increasingly comprehensive representation of the mammalian immune system, based on physiological knowledge and susceptible to further experimental testing and validation. We believe that the integrated nature of FIRM has the potential to simulate a range of responses under a variety of conditions, from modeling of immune responses after tuberculosis (TB) infection to tumor formation in tissues. FIRM also has the flexibility to be expanded to include both complex and novel immunological response features as our knowledge of the immune system advances.

## Background

Mathematical models are a natural approach to improve our understanding of complex biological systems, and ultimately enabling us to predict their behavior and control them [1]. In particular, the intricacies and nonlinear nature of the mammalian immune response have attracted considerable attention over the years [2], in no small part due to the role of the immune response in a variety of relevant human conditions. In addition, the existence of a mathematical model allows one to explore the known differences in immunity development between human and non-human species [3] by altering or excluding specific pathways, as dictated by experimental findings. The basic components of interest would in principle include: cellular or cytotoxic responses (i.e., the development of cells from the T-lineage that attack antigens directly), humoral responses (i.e. the endogenous production of antibodies from cells of the B-lineage that bind to the antigen receptor and hasten its removal) and signaling features (mainly, but not exclusively, through the cytokine network) [4].

In general, the development of quantitative models is often based on the selection of features of interest and their description in mathematical form, followed by their functional integration into a model that can be interrogated and/or used to predict features of interest. Such features can then be compared to experimental data. Similar procedures are followed for immune response models, but due to the system’s complexity, modeling and simulation efforts have focused on specific subsets of the system, such as the cellular responses [5-7], humoral responses [8,9] and/or cytokine networks [10,11], while sometimes excluding or simplifying other components from the model. As a general consideration, the development of comprehensive models is difficult and has to contend with the integrated network nature of the system, where the addition of one novel component necessarily requires defining the interactions of the new item with the remainder of the network.

Several modeling formalisms have been used in developing models of the immune system. Historically, these have been mostly categorized as differential equation models or agent-based models. Agent-based models or cellular automata models of the immune response have attracted great interest [12] since very early studies [13] and have been refined and proposed over the years in a manner that is responsive to new knowledge [14,15]. Their greatest strength is their flexibility and relative ease of use [16], which makes them suitable to model very complex systems without having to mechanistically specify the known component interactions. Instead, the system is defined in terms of “computer agents”, which are sets of rules by which individual actors (i.e. populations of cells, or even individual cells) are created, interact and are destroyed. The modeling effort then focuses on monitoring the interactions among agents, which gives rise to complex, sometimes emergent behaviors that, depending on the rule base, can provide a striking similarity to the temporal evolution of the system being represented. Such models can then be used to develop answers to complex problems, including therapy optimization [17,18]. As others have pointed out [2], despite their power, challenges remain with agent-based models, including the availability of widely accepted software and of model checking and goodness of fit strategies that resemble those commonly used for differential equation models. Differential equation models have provided tremendous insight in the dynamics of complex immunological networks [19,20] and are still widely used, relatively easier to communicate and more readily shared than agent-based models. Some of these models can achieve remarkable degrees of complexity and realism [21]. In addition, differential equations form the backbone of translational pharmacokinetic-pharmacodynamic (PK-PD) models [22,23], the class of models that describe how drug dose influences response through quantitatively linking the drug dose to exposure (pharmacokinetic [24,25]) and the exposure to response (pharmacodynamic [26]) in a living system. These historically are the models of choice in drug research and development.

The integration of multi-scale, realistic models of physiology with pharmacotherapeutic models is a desirable goal that would allow more mechanistic, predictive and overall useful models for drug research and development [27], in addition to enhancing collaborative efforts between biology and modeling. This effort is receiving renewed attention through the area of “systems pharmacology” [28], as explored in two successful interdisciplinary workshops hosted by the National Institutes of Health in 2008 (http://meetings.nigms.nih.gov/?ID=3447) and 2010 (http://meetings.nigms.nih.gov/?ID=8316). Issues related to efficient model sharing and model construction are also the purview of the Interagency Modeling and Analysis Group (IMAG) (http://www.imagwiki.nibib.nih.gov). Building these models accurately and efficiently represents a significant challenge. This has prompted the development of sophisticated software to facilitate integration of separate submodels [29] and parallel computation [30].

The recent availability of computational environments to functionally connect submodels without having to write ad hoc computer code is well complemented by the development of approaches to supervised “monolithic” [30] model integration. In our case, a differential equation framework was chosen for the development of an integrated immune response simulator, coupled with a useful framework, found in systems biology, for integrating multiple subset models into a coherent whole. In this framework, connectivity matrices are built to describe the global network structure, followed by introduction of rate laws to seamlessly integrate multiple biological processes [31,32].

The Fully-integrated Immune Response Modeling (FIRM) simulator is a differential equation based integration of multiple existing models of the immune system [5,8,10]. It accounts for both the humoral and cellular immune response systems and attempts to parsimoniously represent the spatially distributed nature of the system. The goal of this integrated model is to specify antigen exposure over time and calculate predicted antibody levels and cell concentrations following biological perturbations such as immunization or infection. To develop FIRM, we used a pharmacokinetic / pharmacodynamic modeling approach to combine previously published individual models of humoral and cellular response with antigen exposure. FIRM includes both the antigen-specific antibodies and cell populations, and accounts for cytokines and adjuvant components as needed. It is a hybrid construct incorporating structures and parameter values from published models in multiple mammalian species.

This report outlines the stepwise integration of networks describing the cellular dynamics for both T and B-cell responses to bacterial infections and to tumor growth in a target organ. In addition, to illustrate the incorporation of novel mechanisms, we propose and integrate within the framework a new hypothesized model of regulatory T-cell kinetics accounting for immunoevasion.

## Methods

### Model formulation and content

The process of building an integrated simulator starts with the definition of the underlying physiological structure. This preliminarily defines the existing interrelations among all the variables of interest as a “superset” of cellular and molecular populations and reactions. Second, all the cellular and molecular state variables are identified and the interrelationships (transitions) between them determined. The structure of the networks is thereby specified. Third, the mathematical forms of the equations that describe the fluxes are then formulated and their numerical values determined (from literature or existing data). Usually, the first two steps involve the determination and selection of existing relationships that have a physiological basis. As such, they are somewhat easier than the third step, where such relationships need to be made specific and quantitative. The availability of plausible numerical values is a well-known rate limiting step in the definition and assembly of kinetic models, and in the rest of this section we will outline the approach we followed to inform FIRM’s parameterization.

### Mathematical formalism

All the models we considered for integration obey the general governing equation to describe dynamics of cell and mass balance models:

$$dx/\mathrm{dt}=S.v\left(x;k\right)$$

where **x** is the vector of state variables (concentrations of various cell types and molecules) and **v** is the vector of fluxes from one state to the next (i.e. transport processes, reaction rates, cellular fate processes, etc., expressed in concentrations per unit time). **S** is a matrix that describes the structure of the network and its topology. Every column in **S** represents a flux and every row represents a state variable. The vector **k** contains the numerical values of the kinetic and physical constants (often, but not necessarily, expressed in units of inverse time). In general, the vector of fluxes **v** is a function of the state variables and the kinetic and physical constants characterizing transport and reaction processes.

**Figure 1:**
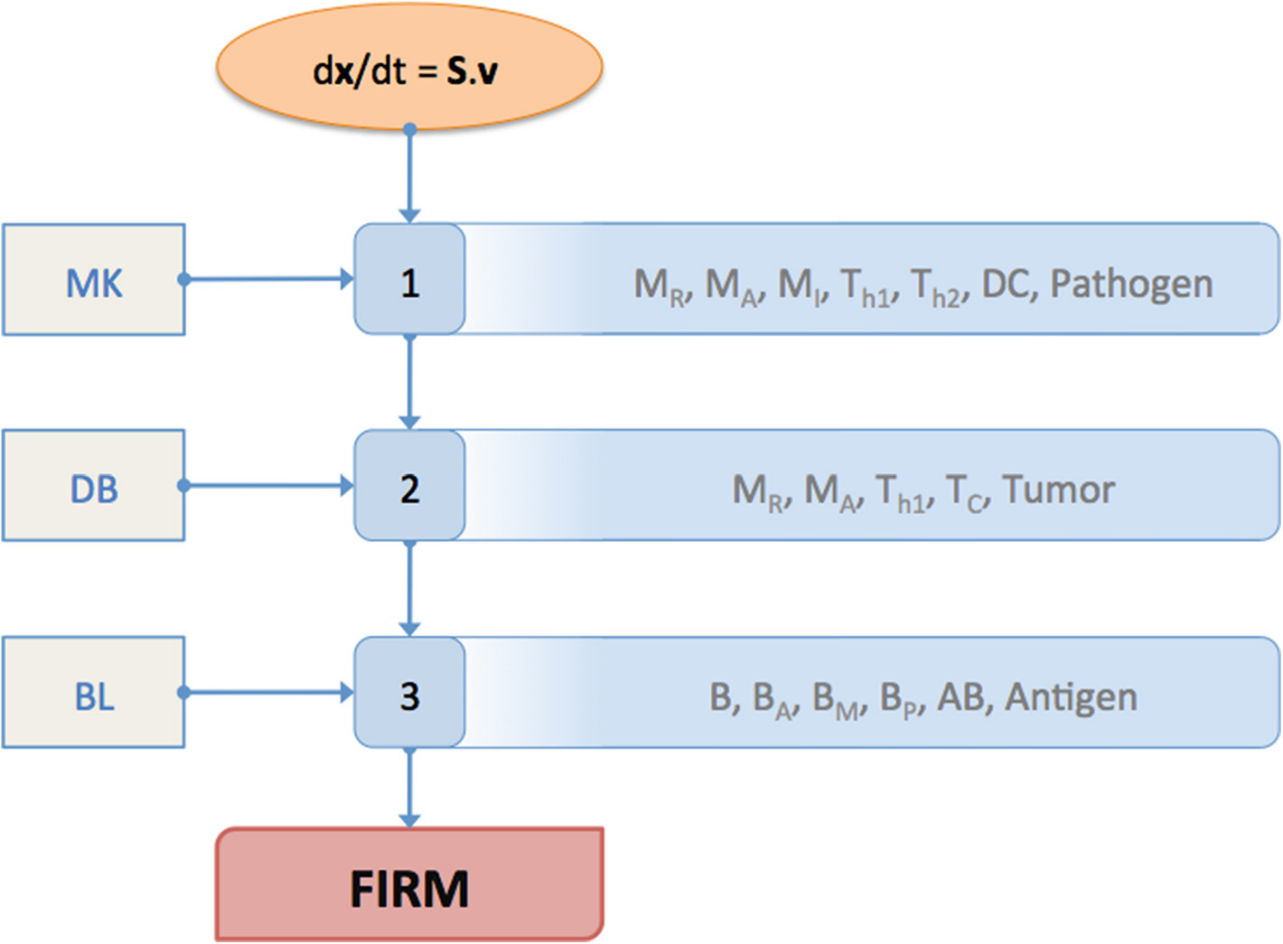
**The reconstructed FIRM network. A**. The final FIRM formulation includes inactive fluxes and nodes that are included for completeness in this figure. Symbols are as follows: TUMOR, tumor mass; DEBRIS, tumor cell debris; MAPC, antigen-presenting macrophages; MA, activated macrophages; MR, resting macrophages; MI, infected macrophages; PI, intracellular bacteria; PE, extracellular bacteria; IDC, immature dendritic cells; MDC, mature dendritic cells; T, naïve T-cells; TCP, cytotoxic precursor T-cells; TC, cytotoxic T-cells; THP, helper precursor T-calls TH1, T-helper 1 cells; TH2, T-helper 2 cells; AB, antibody; B, naïve B-cells; BA, activated B-cells; BM, memory B-cells; BP, plasma B-cells; Treg, regulatory T-cells. See the Supplemental Material for the full details. **B**. The cytokine activity of the FIRM network. Solid green arrows represent production. Dashed green arrows represent up-regulation of a flux, and dashed red arrows represent down-regulation of a flux. The graph is a superset of Figure 1A, where cytokines are superimposed to the previously defined cell populations. Symbols are as follows: I12, interleukin-12; Iγ, interferon-gamma; I10, interleukin-10; I4, interleukin 4; TGF-β, tumor growth factor beta.

Three published models of the immune response, each highlighting different features of the system [5,8,10], were identified for inclusion in FIRM. The reconstructed network for the immune response is shown in Figure 1A for the cell populations involved in the system and in Figure 1B for the cytokines relevant to the model. An explanation of the abbreviations is included in the figure legends. Volume heterogeneity in the model is accounted for and described in the next section. In addition, there were fluxes in the reconstructed network that are inactive in the final FIRM model’s computational (executable) implementation. The reasons for inactive fluxes vary, including for example: redundancy, namely their function is accounted for elsewhere in the model; removal or inactivation of a node; lack of data to properly inform the flux. Full details of inactive fluxes, and the reasons for deactivation, can be found in the Supplemental Material (Additional file 1: Table S8). Since some fluxes were inactivated, not all the nodes we initially considered as part of FIRM were active in the final structure: specifically, the function of M_APC_ (macrophages functioning as antigen presenting cells) is incorporated in the dendritic cell population and not explicitly accounted for; and, the function of T_H2_ in the humoral response was not included due to lack of quantitative information regarding this component. Consequently, the relevant cytokine network components are also inactive.

The matrix **S**, the state variables and the fluxes corresponding to the final structure of the FIRM model are found in supplemental Additional file 1: Tables S1, S2 and S3. The mathematical form of all the flux variables are given in Additional file 1: Table S4, and the numerical values and their literature sources are found in Additional file 1: Table S5 and S6.

### Spatial distribution features

To account for known features of the spatial distribution of the immune response components in our simulations in a parsimonious manner, the FIRM model has five separate tissue spaces where the cell and cytokine populations can travel: lung (assumed to have a volume of 1000 mL), blood (4500 mL), lymphoid tissues relevant to the cellular (10 mL) and humoral response (150 mL) and the sites of immune recognition (4500 mL). As populations of cells and molecules travel between biological spaces, their concentrations are multiplied by the respective volumes of distribution so as to maintain mass balance. This was particularly important for the population of infected macrophages, which changes dynamically as bacterial infection progresses and turns out to have a time-dependent variable volume of distribution whose features needed to be accounted for in the simulation. Additional file 1: Table S7 in the Supplementary Material contains the various tissue space volumes.

It is worth noting that this is a parsimonious representation since, to accurately represent spatially differentiated behaviors, one would have to define biological spaces for each spatially (and functionally) separate component of each organ or tissue that has a distinct pattern or behavior from other parts of the organ or tissue under consideration. These spaces would be defined so as to have different volumes, rate constants for accessibility, etc., to reflect their heterogeneous physical structure and, given the number of parameters required, would require detailed experimental information at the cellular and molecular level. In this sense, FIRM is a parsimonious model and does not reach this level of granularity, although the model structure is amenable to be extended and incorporate such considerations where warranted, required by the purpose of the modeling exercise and supported by the data.

### Simulation platform and integration procedure

**Figure 2:**
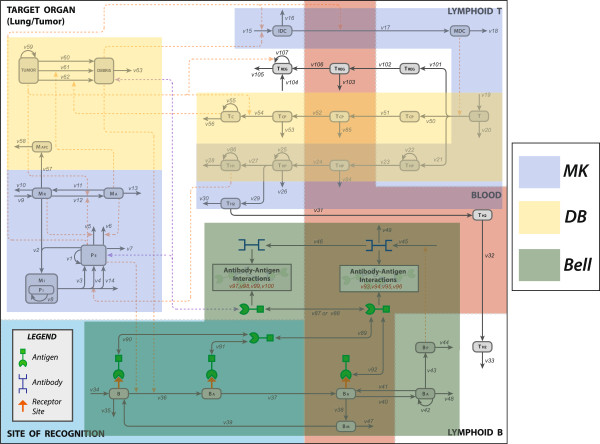
**FIRM integration formalism.** This figure summarizes the plan devised for the development of FIRM. The oval at the top represents the method of building dynamic models that was employed to construct FIRM. At each step, the subset model included can be seen on the left, and the major cell populations covered in each step is outlined on the right. MK refers the Marino-Kirschner model (including resting macrophages M_R_, activated macrophages M_A_, infected macrophages M_I_, T-helper 1 cells T_h1_, T-helper 2 cells T_h2_, dendritic cells DC and pathogen [10]), DB refers to the DeBoer et al. model (including resting macrophages M_R_, activated macrophages M_A_, T-helper 1 cells T_h1_, cytotoxic T-cells T_C_ and Tumor [5]), and BL refers to the Bell model (which includes naïve B-cells B, activated B-cells B_A_, memory B-cells B_M_, plasma B-cells B_P_ and Antigen [8]). “Pathogen” indicates bacterial infection, while “Antigen” refers to viral or other small antigen infection. The partial overlap of the models provides a roadmap to integration, which however needs to take into account the diversity of formulations used in the models to account for essentially the same immune response features.

Mathematica (Wolfram Research, Champaign, IL) was used as the model-building platform. The integrated model was assembled in a stepwise fashion. Specifically, the concentration and flux vectors in the model structure were populated step-by-step with the appropriate features and components, as illustrated in Figure 2. FIRM was built in Mathematica 7.0. The Mathematica workbook that resulted was used to generate all graphs in this paper from FIRM simulations. The simulator is portable to other simulation platforms: a Matlab (The Mathworks, Natick, MA) version of the FIRM simulator was also generated. Additional file 1: Tables S1 to S6 contain a full specification of the FIRM simulator, suitable for implementation in other matrix languages.

## Results and discussion

### (1) Procedure for integrating multiple dynamic models from different sources

As described in the Methods section, the dynamic simulator is expressed as a series of balance equations. Mathematically:

$$dx/\mathrm{dt}=S.v\left(x;k\right)$$

where **x** is the vector of state variables (cell types and molecule concentrations) and **v** is the vector of fluxes from one state to the next (i.e., transport processes, reaction rates, cellular fate processes, etc.). **S** is a matrix that describes the structure of the network and its topology. Every column in **S** represents a flux and every row represents a state variable. The vector **k** contains the numerical values of the kinetic and physical constants. The contents of all these mathematical objects are found in Additional file 1: Tables S1 (Stoichiometric matrix), Additional file 1: Table S2 (variable list), Additional file 1: Table S3 (flux list), Additional file 1: Table S4 (rate laws), Additional file 1: Table S5 (kinetic constant values), and Additional file 1: Table S6 (miscellaneous parameter values).

The reconstructed network is shown in Figure 1A. It includes all the major T and B-cell types, the pathogens (antigens), as well as tumor and its debris. The regulatory effects of the major inflammatory growth factors and cytokines are shown in Figure 1B. These graphs highlight the boundaries of FIRM. The processes that we considered for inclusion have been described multiple times (see e.g. figure in [33] for a description) and relate to humoral and adaptive immunity. Briefly, the humoral side of the system describes the activity of B-cells that, when in contact with an antigen/pathogen, secrete antibodies which are essentially a secreted version of their receptor compatible with the antigen). The antibodies bind to the antigens and neutralize them. The link between the humoral and the cellular components of the system is provided by helper T-cells, which activate B-cells. This function is currently not included in the model since it was not a part of the constituent submodels. The cellular side starts with naïve T-cells recognizing antigen epitopes through antigen presentation via dendritic cells and macrophages and subsequently developing a T-cell driven response to the antigen. This process is particularly important when the pathogen is a cell, such as in cancer and bacterial immunity, which are both described in FIRM. T-cells can also differentiate to regulatory T-cells, which essentially mute features of the immune response.

The FIRM simulator was built in a step-by-step fashion, summarized in Figure 2, from both constituent models that have appeared in the literature [5,8,10] and novel mechanisms. A summary of those steps is below.

**Figure 3:**
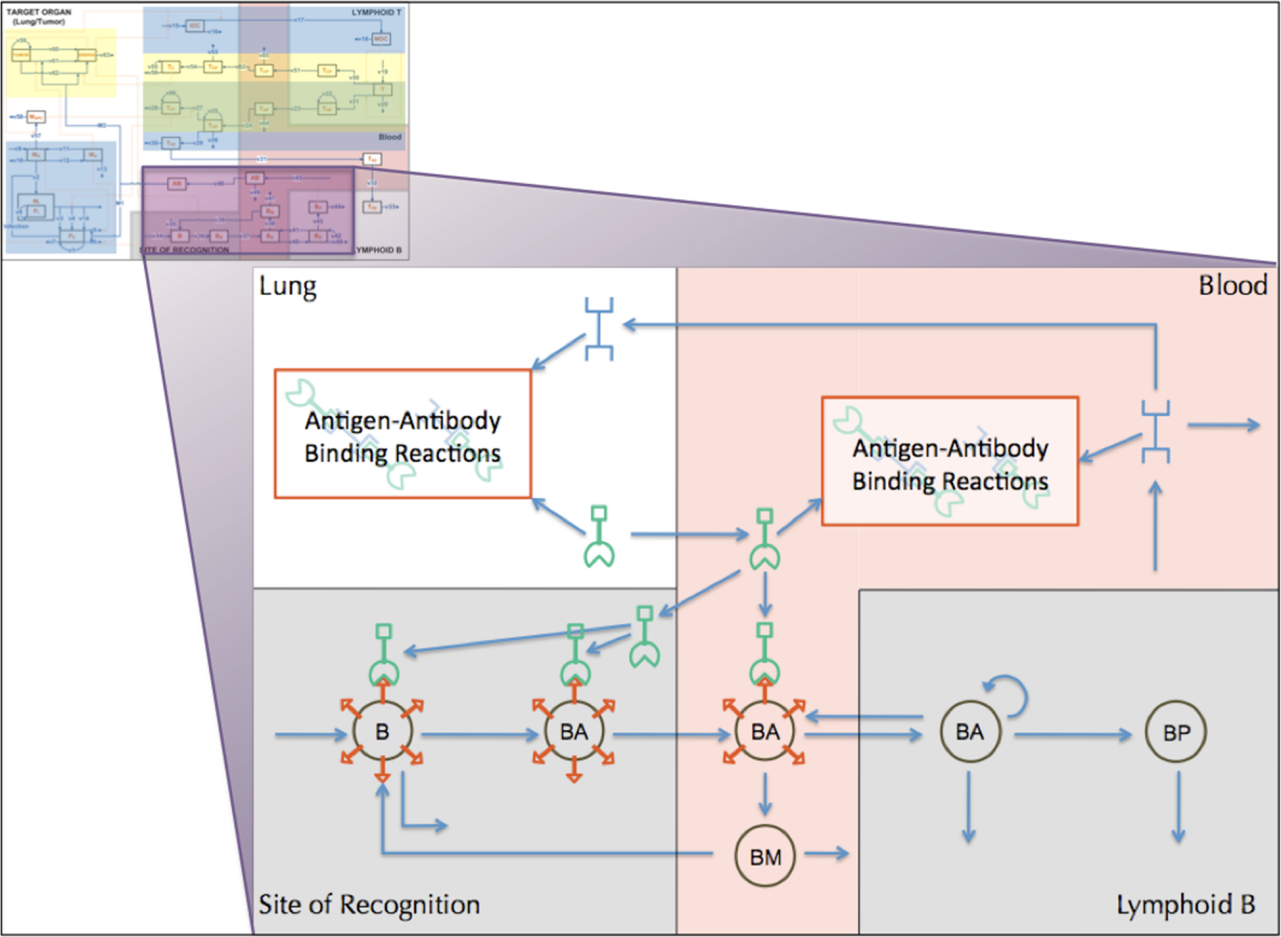
**The individual areas of influence of the three original models (MK, DB and BL) in relation to the FIRM network structure.** There was overlap in the content of the original models, exemplified here by the overlapping shaded areas of the MK ([10]) and DB ([5]) models (light green). Nodes not encompassed by a shaded area are inactive in the final FIRM structure but have been identified as connections among models and are reported for completeness. See the Supplemental Material (Additional file 1) for full details on inactive fluxes and nodes.

*Immune response to tuberculosis (TB) infection*[10]*: Cellular response to bacterial challenge.* The model integration in FIRM started with a published model for TB infection of the lung (by Marino and Kirschner, hereafter the MK model). This model described the activation of macrophages, their infection, and the antigen presentation by dendritic cells that leads to differentiation of T-cells in lymphoid tissue; these cells then migrate to the lung where they differentiate into T1 and T2 helper cells. The scope of this subset model is described in Figure 3. The MK model also included a rather detailed representation of the cytokine signaling network following infection, which is not shown in Figure 1A for simplicity (but is shown in Figure 1B).

The MK model state variables and fluxes were introduced into the network and used to specify the x and v vectors in the overall mass-balance model. This was done in a step-wise fashion and the process was quality controlled at each step. Briefly, to ensure quality control of the implemented model, all fluxes in the network are turned off except one at a time (the one that needs to be examined) and conservation of mass is checked for. This is repeated every time a new population of either cells or molecules is introduced in the model, thus ensuring that no arbitrary gains or losses occurred at any step during model building. A sample QC/QA document is provided in Additional file 1: Figure S10. There were several issues and simplifications associated with mapping the MK model onto the unified network structure at the basis of FIRM. These included changes in basal states (which are calculated analytically as functions of parameter values), accounting of cell population dynamics to obey mass balance (specifically, macrophages and bacteria) and accounting for the variable volume of distribution of the infected macrophages and for the appropriate volume ratios for cell and molecule migration. As a final check, the simulations of this initial model were compared with those available from the original publication (Additional file 1: Figure S9). While the agreement was not exact, this was to be expected given that changes were made to the original model formulation. Details regarding the mapping of the MK model onto the FIRM framework are provided in the Table 1.

**Table 1:** Overall summary of integration issues

**Integration issues resolved with the mapping of the MK model [Ref.**[10]**] onto the FIRM network structure**	**Integration issues resolved with the integration of the DB model [Ref.**[5]**] with the MK model [Ref.**[10]**]**	**Integration issues resolved with the integration of the BL model [Ref.**[8]**] into the FIRM framework comprised of the DB model [Ref.**[5]**] and the MK model [Ref.**[10]**]**
• Added basal state levels of resting macrophages and IDC using MK latency parameter values:	• Removed “HTL” (T_H1_) from activation of macrophages.	• B_M_ conversion to B cells has the same rate as the death of B_M_.
- M_R_[0] = 5 * 10^8^ cells	• Using DB value for M_A_ half-life.	• Antibody produced by B_P_, B_A_ in blood, and B_A_ in lymphoid B.
- IDC [0] = 5 * 10^7^ cells	- η_13_ = 1 day^-1^	• Expansion of the BL model due to the relaxation of equilibrium assumptions required the creation of variables x_41_-x_52_ and fluxes v_87_-v_100_.
• Combined bursting (v_3_) and natural death (v_14_) of infected macrophages into one flux (v_3_). The reaction rate will be the summation of the two individual reaction rates.	• Created constant recruitment of T_CP_ and T_HP_ in the lymphoid T, in fluxes v_50_ and v_21_, respectively, analogous to I1 and I2 from the DB model.	• Created “antigen” variable in blood and Site of recognition.
• Introduced M_I_ (Infected Macrophages) as a separate space with a variable volume:	- I1 → ρ_50_ , I2 → ρ_21_	- “Antigen” can be either a tumor debris or a bacteria cell, they become a part of the “antigen” pool once they enter the blood
- Volume_MI_ = 8*10^-9^ * x_3_	• Accounted for T_CP_ presence in the blood, created a separate variable x_25_.	- Permeates from lung to blood, and from blood to Site
• Fixed bacteria accounting issues:	• Temporarily changed HTL (T_H1_) in FACTOR to HTLP (T_HP_).	
- In the MK model, half of the amount of bacteria released during bursting was required to infect one macrophage. These two amounts have been made independent, but they are currently set to 25 bacteria for infection and 50 bacteria for bursting. These are the same values used in the MK model, but they can be changed easily.	• Using DB value for M_R_ half-life.	• Created receptor sites on select B cell populations.
- Bursting (v_3_) is based on the ratio of intracellular bacteria to infected macrophages (x_5_/x_3_). The bursting will occur at a greater rate as the ratio approaches a set number (the macrophage’s capacity). This capacity is currently set to 50.	- η_10_ = 0.05 day^-1^	- x_16_, x_17_, and x_18_ have receptor sites
- Bursting (v_3_) releases x_5_/x_3_ extracellular bacteria into the system, instead of a fixed number.	• Redefined FACTOR with HTL (T_H1_).	- Receptor sites have 2 states: antigen-bound and free
- T-cell induced apoptosis (v_4_) releases x_5_/x_3_ extracellular bacteria into the system.	• Added T_H1_ (HTL) proliferation from the DB model as a negative term to the death flux v_28_.	• Expanded antigen-B cell interaction to include receptor sites and binding events.
- It is important to note that x_5_/x_3_ is a time-dependent ratio.	• Modeled differentiation of naïve T cells to T_CP_ (v_50_) to mirror the action of v_21_ from the MK model.	- All antigen-receptor binding events occur at the same rate
• Combined naïve T cell death and recirculation from the MK model into one clearance flux (v_20_). The reaction rate will be the summation of the two individual reaction rates.	• Modified M_APC_ from the DB model. M_APC_ and its corresponding fluxes (v_57_, v_58_) will remain inactive and undefined. The functionality of M_APC_ described in the DB model, using the variable INFLAM, will be merged with the dendritic cells:	- The receptor-antigen binding event is a reversible reaction
• Added basal state levels of resting macrophages and IDC using MK latency parameter values but using the new clearance flux of naïve T cells (v_20_):	- Added term to recruitment of IDC cells (v_15_) using INFLAM as a trigger.	- x_20_ is assumed to have the same receptor state as x_18_
- T[0] = 98,039 cells	■ (ρ_21_ + ρ_50_)/2 * INFLAM	• Expanded antigen-antibody interactions to include dynamic single- and double-bound states.
• Modified rate law v_22_. The MK formulation allowed for negative proliferation.	- Added term to migration/maturation of IDCs (v_17_) using INFLAM as a trigger.	- All antigen-antibody binding events occur at the same rate
• Accounted for T_HP_ presence in the blood, created a separate variable x_13_.	■ Used term from MK stimulation, but replaced x_4_ (P_E_) with INFLAM	- All bound antibody states are cleared at the same rate
• Used volume ratios to properly account for cell migrations across tissue space borders.	• Cut off an INFLAM feedback loop by globally redefining INFLAM without HTL (T_H1_) when substituting in FACTOR. Now, the only variable that determines INFLAM is tumor burden. The basic premise of the INFLAM loop is an increase in INFLAM causes dendritic cells to produce more helper T cells, and the creation of these helper T cells caused FACTOR to increase, which in turn caused INFLAM to increase.	- Binding events occur in both the blood (with “antigen”) and the lung (with extracellular bacteria)
• Eliminated flux v_30_. The migration flux of T_H2_ to the blood (v_31_) that was to be added with the B cell response will take its place. v_31_ will take the death rate of v_30_ (0.3333 day^-1^) as its reaction rate. Having two fluxes drain the T_H2_ population was leaving the T_H2_ levels in the lung much too low and ineffective.	• Added new fluxes to FIRM structure:	• Defined initial conditions with analytical solutions for B cells and B cell free receptors sites.
• Added basal state levels of IL-12 in the lung, produced by the basal levels of M_R_.	- v_84_ → death of T_HP_ in the blood	• Permeation of tumor debris to blood is turned off.
IL-12[0] = 5*10^8^ (q_78a_/η_79_)	- v_85_ → death of T_CP_ in the blood	- Tumor-antibody interaction lacks definition at this time
	- v_86_ → proliferation of T_H1_ in the lung (removed negative term from v_28_)	
	• Defined initial conditions with analytical solutions for:	
	- M_R_, IDC, T, T_HP_ in the lymphoid T, T_HP_ in the blood, T_CP_ in the lymphoid T, T_CP_ in the blood, IL-12 in the lung	

*Immune response to tumor formation*[5]*: Cellular response to tumor challenge.* The second subset model identified for inclusion in the integrated model (by DeBoer et al., hereafter the DB model) described the inflammation response to the presence of a tumor. Its components were the growth of tumor mass, the activation of macrophages in response to the tumor cells, the proliferation and differentiation of cytotoxic and T1 helper cells, and the killing of tumor cells creating tumor debris. The original formulation of this subset model had all tracked populations in a single biological space; therefore, the cell populations described in the model had to be mapped to their appropriate organs. The scope of this model can be seen in Figure 3, together with its overlap and points of contact with the MK model. Once again, the state variables and fluxes associated with the content of this subset model were added to the model network.

The inclusion of the DB model marks the first integration in the FIRM system of two separately developed and reported kinetic models (MK and DB). The integration of two kinetic models resulted in some complexities, reflecting in turn: the state of biological knowledge revealed by the models, the assumptions made, the structure of the network, and the detail of the quantitative information. The process of integrating models from various sources and built for different mammalian species requires explicit and sometimes implicit biological and structural assumptions. We summarized those choices as “integration issues” and they are reported in detail in the Table 1. The resolution of integration issues is critical to the construction of an integrated model such as FIRM. For example, the DB model included processes and parameters which tended to be descriptive as opposed to mechanistic, reflecting the knowledge of immunology at the time. Therefore, the issues involved with the addition of the DB model included integrating phenomenological parameters into the FIRM state variable and rate law structure (and sometimes modifying them), selecting a value for kinetic constants that appear in both models, and including new network fluxes, mostly cellular, as necessary to integrate the models to ultimately ensure balance of mass.

*B-cell response to antigen*[8]*: Humoral response to antigen challenge.* The third subset model that was included in FIRM (by Bell, hereafter the BL model) details the B-cell and antigen response to the presence of an antigen in the system. Included in the BL model is the exposure of naïve B-cells to antigen, the activation of B-cells that migrate to the lymphoid B via the blood, the differentiation of activated B-cells to plasma and memory B-cells, and the production of antibodies by said B-cells. Lastly, the antibodies work to eliminate the antigen from the blood and the target organ. This model’s components are again shown in Figure 3, which also highlights commonalities with the MK and DB models.

**Figure 4:**
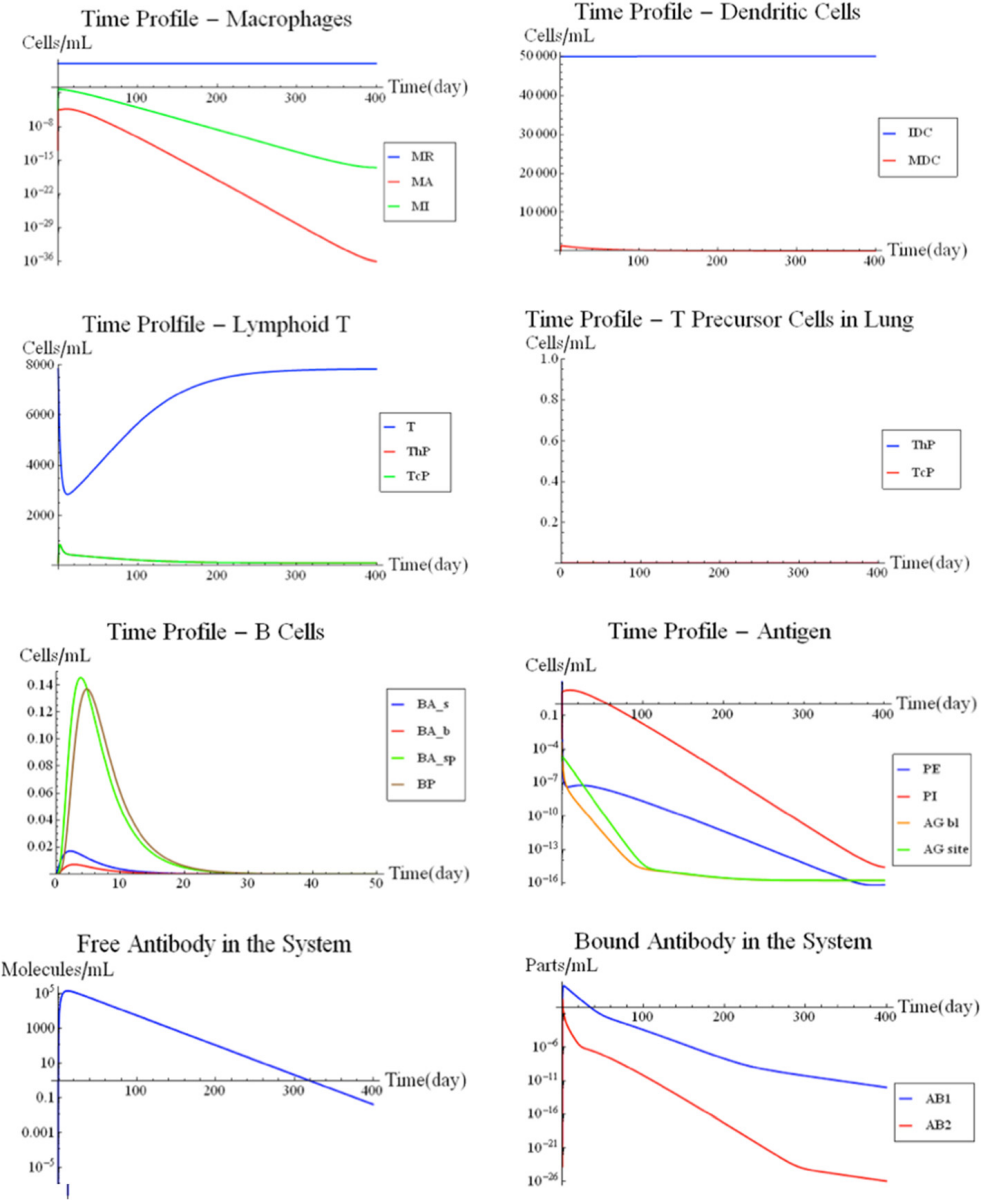
**Scope and details of the BL model in the context of the FIRM network.** The model [8] included detailed information on the interactions of antigens, antibodies, and B-cell receptor sites of the humoral response. Symbols are as follows: B, naïve B-cells; BA, activated B-cells; BP, plasma B-cells; BM, memory B-cells. Bivalent antibodies are released by BA and BP in the lymphoid B organs and bind antigens both in blood and lung (target organ) tissues. The antigen binds to naïve and activated B-cells and stimulates the formation of antibody.

Again, the integration of the BL subset model into the framework of FIRM led to some integration issues. The BL model had a rich level of molecular detail when describing the interaction of B-cell receptors, antibodies, and antigens. These interactions were simplified in the original model through the use of quasi-equilibrium assumptions. Given the level of granularity in FIRM, these assumptions could be relaxed and the full details of the underlying molecular processes are described. Therefore, the network structure accommodates antigen and antibody binding (Figure 4). The inclusion of this subset model in the network was a major integration issue with FIRM, since the network had to be substantially expanded to include all of these detailed processes. Integration issues are reported in detail in the Table 1. Briefly, antigen-antibody binding reactions (shown in Figure 4) include: a free antibody binding to a free antigen creating a single-bound antibody; a single bound antibody binding to a free antigen creating a double-bound antibody; the removal/clearance of a single-bound antibody; and the removal/clearance of a double-bound antibody.

**Figure 5:**
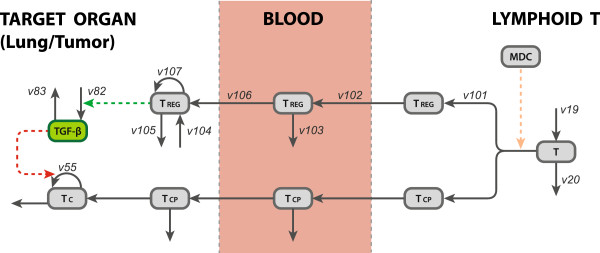
**Cellular and Humoral Response to Antigen Presentation.** The cellular (upper panel) and humoral (lower panel) response of the fully-integrated FIRM simulator with nominal parameter values and an initial load of 100,000 antigen molecules in the target organ, which are allowed to infect macrophages and migrate into the blood. While the cellular response is small and has little to no effect on the infection, the humoral response is strong and effectively eliminates the infection.

The incorporation of the BL model essentially completed the FIRM structure based on published models. Figure 5 shows a simulation of the fully integrated FIRM model at nominal parameter values (Additional file 1: Tables S5 and S6). This is a base case simulation with an initial load of 100,000 infecting bacterial cells in the target organ. The bacteria cells are allowed to migrate into the blood as well, thus triggering a strong antibody response. The B-cell receptor density for the bacteria is assumed to be 10^3^ molecules/mL, in keeping with the BL model.

It should be mentioned at this point that the constituent models composing FIRM were not necessarily tailored to a particular animal species. The BL model description mentions data being obtained in rabbits, but it also states that the model recapitulates essential features of the mammalian immune response. The DB model is based on data in mice, while the MK model was built to be applicable to humans. This makes FIRM a hybrid model containing features of a few mammalian species. That being said, validation of models against data is a tool of paramount importance in model development. FIRM provides predictions of timing and extent of immune respone, which can be compared against experimental data. While we propose here the FIRM structure and have strived to maintain consistency of units across the models, we make no attempt at validation and later we propose approaches to test this important question.

*Addition of* T_reg_*and* TGF-β: With the integration of the MK, DB, and BL models, we have created a platform with which basic immunological simulations can be performed. The FIRM platform is flexible enough to easily introduce new information and physiological responses. This section demonstrates that principle through addition of regulatory T-cells (T_reg_). T_reg_ play an important role in the immune system’s response to a tumor. T_reg_ are a rather new discovery in the immunological field, and considered to be of great importance. Therefore, T_reg_ were selected to be the first addition when expanding the FIRM platform beyond the original publications.

**Figure 6:**
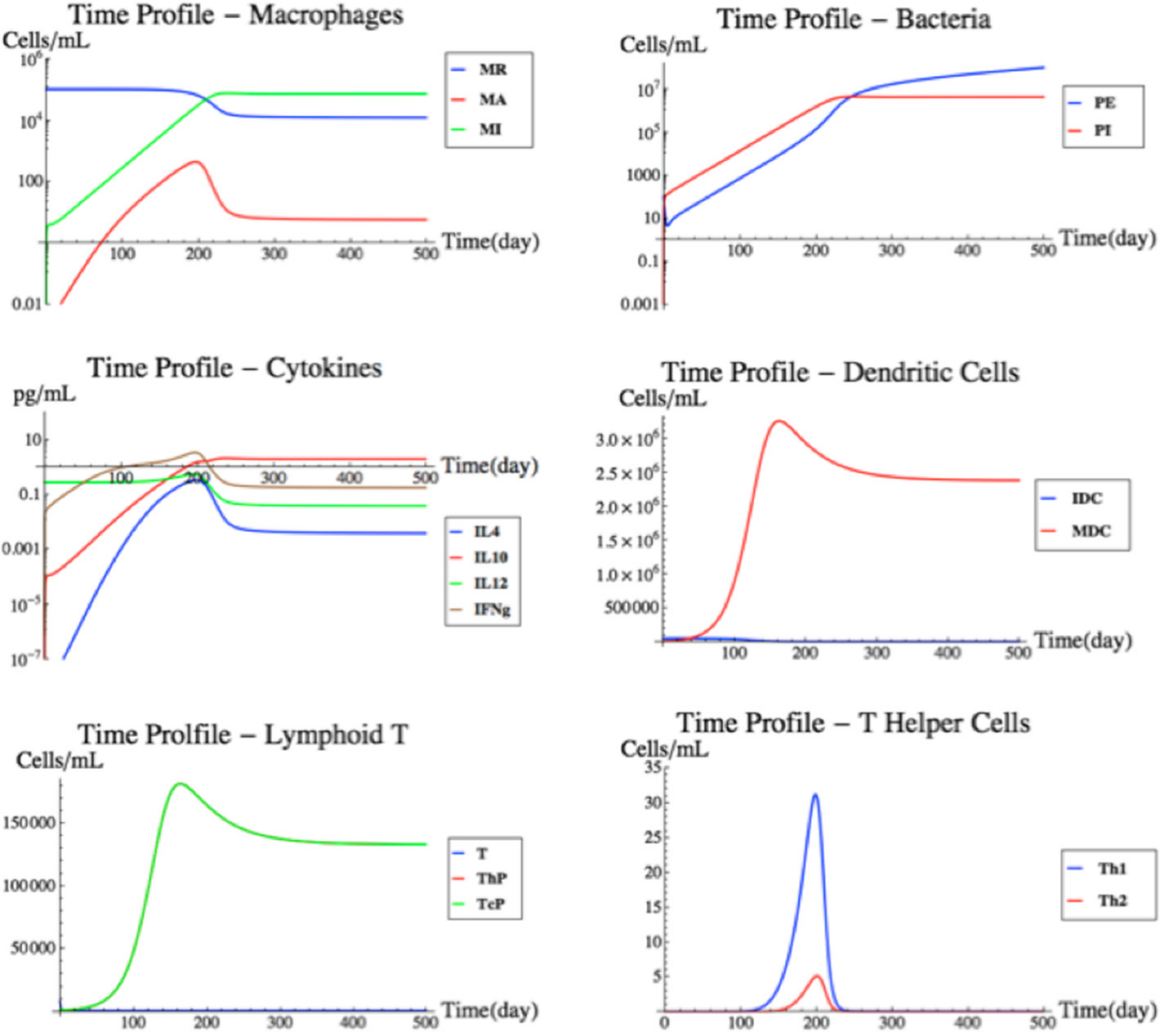
**The T**_**reg**_**kinetic model incorporated in FIRM.** The model accounts for T_reg_ presence in the lymphoid T (where they differentiate from naïve T-cells), the blood (where they migrate and are subject to removal or recruitment to the target organ) and the target organ (where they proliferate). The kinetics of TGF-β are similarly accounted for. T_reg_, regulatory T-cells; TGF-β, tumor growth factor beta; other abbreviations as in Figure 1A and Figure 1B. See text for details.

There are two sources of T_reg_ in the body: the first source is from the lymphoid T and the second source is a resident population in the target tissue [34]. T_reg_, much like the T_CP_ and T_HP_, will also have a constant differentiation from the naïve T-cell population in the lymphoid T that will travel into the blood. T_reg_ account for 5%-10% of the T-cells in the blood in a normal state [34]. The differentiation in the lymphoid T was calibrated appropriately to reflect these levels. Once the T_reg_ reach the site of the tumor, they produce TGF-β, a cytokine that has a down-regulatory effect on the proliferation of cytotoxic T-cells. Cytotoxic T-cells assist in killing tumor cells, so it stands to reason that tumor cells play a role in the proliferation of T_reg_ in the target tissue. A visual representation of the T_reg_ addition can be seen in Figure 6. T_reg_ exist in the lymphoid B, the blood and the target organ. Naïve T-cells differentiate to T_reg_ in the lymphoid T; they migrate to the blood where they are subject to removal or recruitment to the target organ; they can also proliferate in the target organ. TGF-β is produced and decays in the target organ, where it exerts its effect on T-cell kinetics. The majority of rates and parameters used in the rate laws were assigned values based on approximations. These approximations were picked to be similar to the values of the rates associated with other T-cell lineages and cytokines in FIRM. The exact numerical values of the rates and parameters are still a subject of evaluation and examination of relevant literature and future experimentation.

*The basal state:* As with all kinetic models, FIRM has a basal (unperturbed) state. In this particular case, the basal state represents the immune system components’ baselines when there is no antigen present. Such a basal state reflects steady state homeostasis and was calculated using the nominal parameters and running the model to stable steady state conditions. Calculated basal state cell populations include: 10^8^ resting macrophages in the target organ, 5 × 10^7^ dendritic cells in the target organ, ~8 × 10^4^ naïve T-cells in the lymphoid T, ~10^3^ T helper precursor cells in the lymphoid T, 3 × 10^3^ T helper precursor t cells in the blood, ~2 × 10^4^ naïve B-cells in the site of recognition, ~10^3^ cytotoxic T precursors in the lymphoid T, 5 × 10^4^ cytotoxic T precursors in the blood, ~3 × 10^2^ molecules of IL-12 and ~3 × 10^2^ of TGF-Beta in the target organ, 10^4^ T_reg_ in the blood, 2 × 10^2^ in the lymphoid T and 10^6^ in the target organ, and ~2 × 10^7^ free receptor sites on the naïve B-cells in the site of recognition. The T_reg_ in the blood account for about 9.1% of all T cells circulating in the blood [34]. In the basal state, the system is free of antigens, and, therefore, antibodies. This basal state can then be perturbed by exposure to antigen reflecting various stimuli or pathological situations. The response to one or many antigens can be simulated.

The final version of the FIRM simulator has 55 nodes (cells and antibodies), 107 distinct processes and 171 parameters. As defined and with the postulated interactions and parameter values described above, the FIRM simulator is an initial step towards a simulator of the immune response capable of representing a variety of putative challenges. To demonstrate the use of FIRM we present four case studies that illustrate its different features and capabilities.

### (2) Use of the simulator – case studies

Based on the full model, changes in network structure and parameter values can be defined to mimic known occurrences in immune response modulation. Within FIRM, completely different situations involving immune system cells, and foreign and endogenous molecules, can be modeled in a few steps. Deactivating one or two fluxes can drastically change the conditions of a simulation. Among many possible simulations, four cases of interest are explored:

1. TB infection;

2. Blood borne pathogen infection;

3. Spontaneous tumor rejection;

4. Influence of T_reg_ on tumor rejection.

The first two case studies represent confirmatory simulations for comparison with the original MK and BL model results.

### TB infection

To simulate a pure TB infection, 100,000 extracellular bacteria were introduced into the lung (target organ) and flux v_87_ was deactivated. Flux v_87_ represents the permeation of extracellular bacteria between the target organ and the blood (Figure 1A). Flux v_87_ was thus deactivated to prevent the bacteria from migrating away from the lung. The TB infection is known to stay local in the lung and not permeate into the blood. FIRM was then simulated with its nominal parameters.

**Figure 7:**
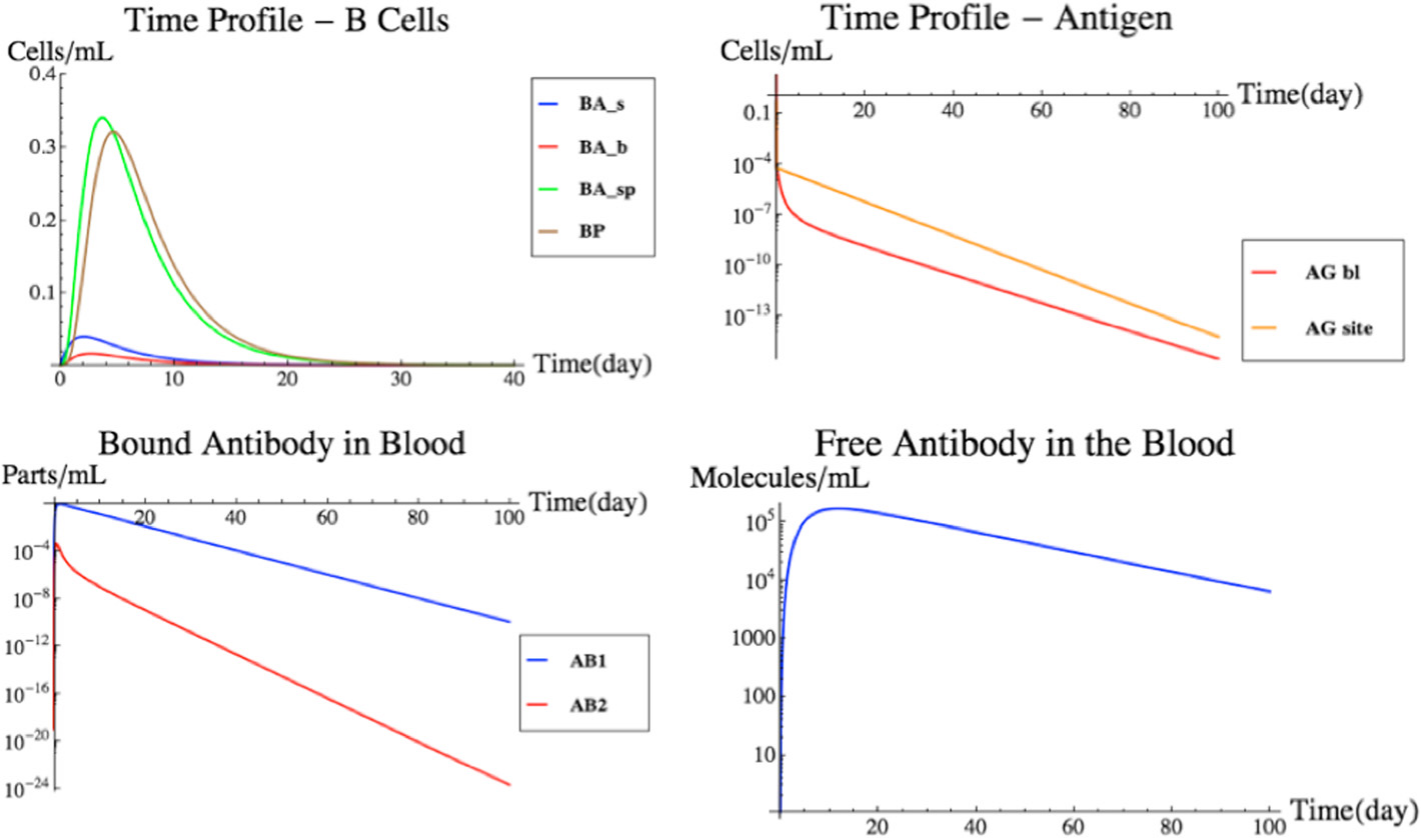
**TB infection simulation.** A simulation of an intracellular bacterial infection with an initial condition of 100,000 bacteria in the target organ. See text for details.

As shown by the MK model, a TB infection is thought to be dealt with exclusively by the cellular response with no response from the humoral system. A chronic infection is simulated. The T-cell response that is solicited by the immune system is enough to prevent runaway growth by the bacterial population in the lung. Even though the cellular response prevents runaway growth, the levels of infected macrophages are rather large and stay constant. The results can be seen in Figure 7. As one would expect, the simulation results closely resemble the MK model, which was specifically developed to represent TB [10]. This simulation illustrates that the integrated FIRM can still recapitulate the trends initially found with the MK model.

### Blood borne pathogen

**Figure 8:**
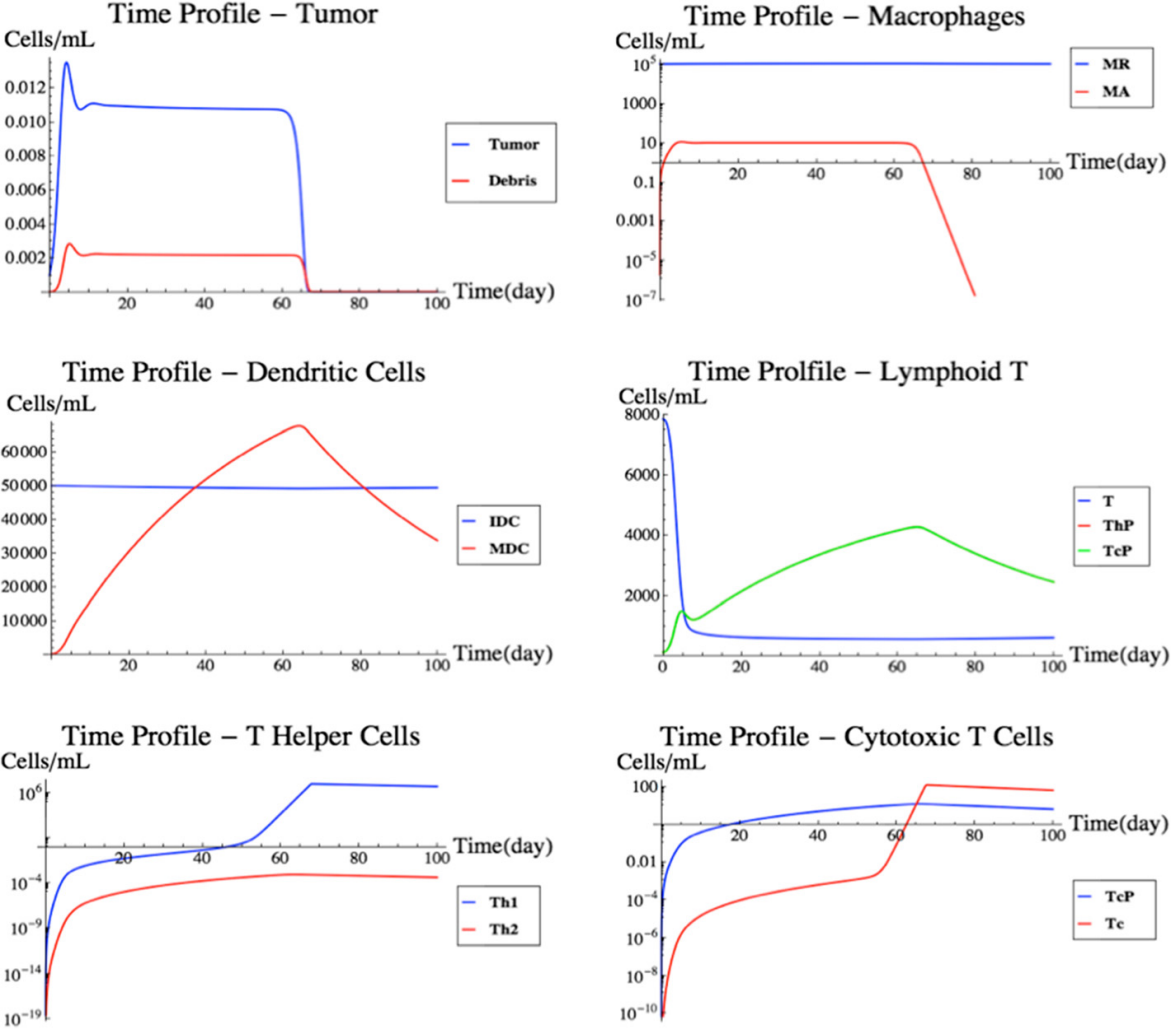
**Blood borne pathogen simulation.** A simulation using nominal parameter values and an initial condition of 100,000 antigen molecules limited to the bloodstream. This simulation is different from that in Figure 5, where the antigen appeared in the target organ. See text for details.

In this simulation, a blood borne pathogen originates in the blood and remains confined within the circulation. For simplicity, the increase in antigen load prior to the induction of an immune response is represented as a spike (pulse) increase. An example of this situation would be a viral infection. The pathogen does not permeate into the tissue to infect, for example, resident macrophages. To simulate a blood borne pathogen in FIRM, fluxes v_87_ and v_88_ were deactivated. These two fluxes model the access of antigen between the target organ and the blood. This simulation used nominal parameter values and an initial condition of 100,000 antigen molecules in the blood. The results of the simulation can be seen in Figure 8.

The humoral (B-cell) response was used by FIRM to eliminate the infection. In fact, in the case study, as expected, the cellular response is virtually non-existent. The antigens never come in contact with the dendritic cells in the tissue. Therefore, there is little antigen presentation to the T-cells in the lymphoid T to drive differentiation of the naïve T-cells. The graphs in Figure 8 show a fast humoral response flooding the blood vessels with antibodies. The antigens are quickly bound to the antibodies in the blood and are removed from the system as antibody-antigen complexes. This simulation resembles the behavior of the original BL model, confirming the performance of the integrated FIRM against the individual submodels.

### Tumor removal

Since no tumor-antibody interaction is defined in FIRM at this point (all the individual models, MK, DB or BL, lacked a mechanistic description of antibody-mediated cell kill), simulations of tumors will not include migration of tumor antigens (debris in the DB model) into the blood from the target organ. Therefore, the flux v_88_ was deactivated for this case study. Additionally, we changed the half-life of all T helper cells to 0.02 day^-1^ from 0.3333 day^-1^ (the remaining reaction rates were left at the nominal parameter values). The updated half-life was derived from the DB model, while the previous half-life was derived from the MK model. As a justification, through successive model runs we observed that an elevated half-life for the T-cells prevented them from encouraging proliferation of cytotoxic T-cells and therefore prevented tumor kill. This shows that tumor removal by the immune system may be influenced by the kinetics of these cells and also points to features of the model that greatly influence its predictions.

**Figure 9:**
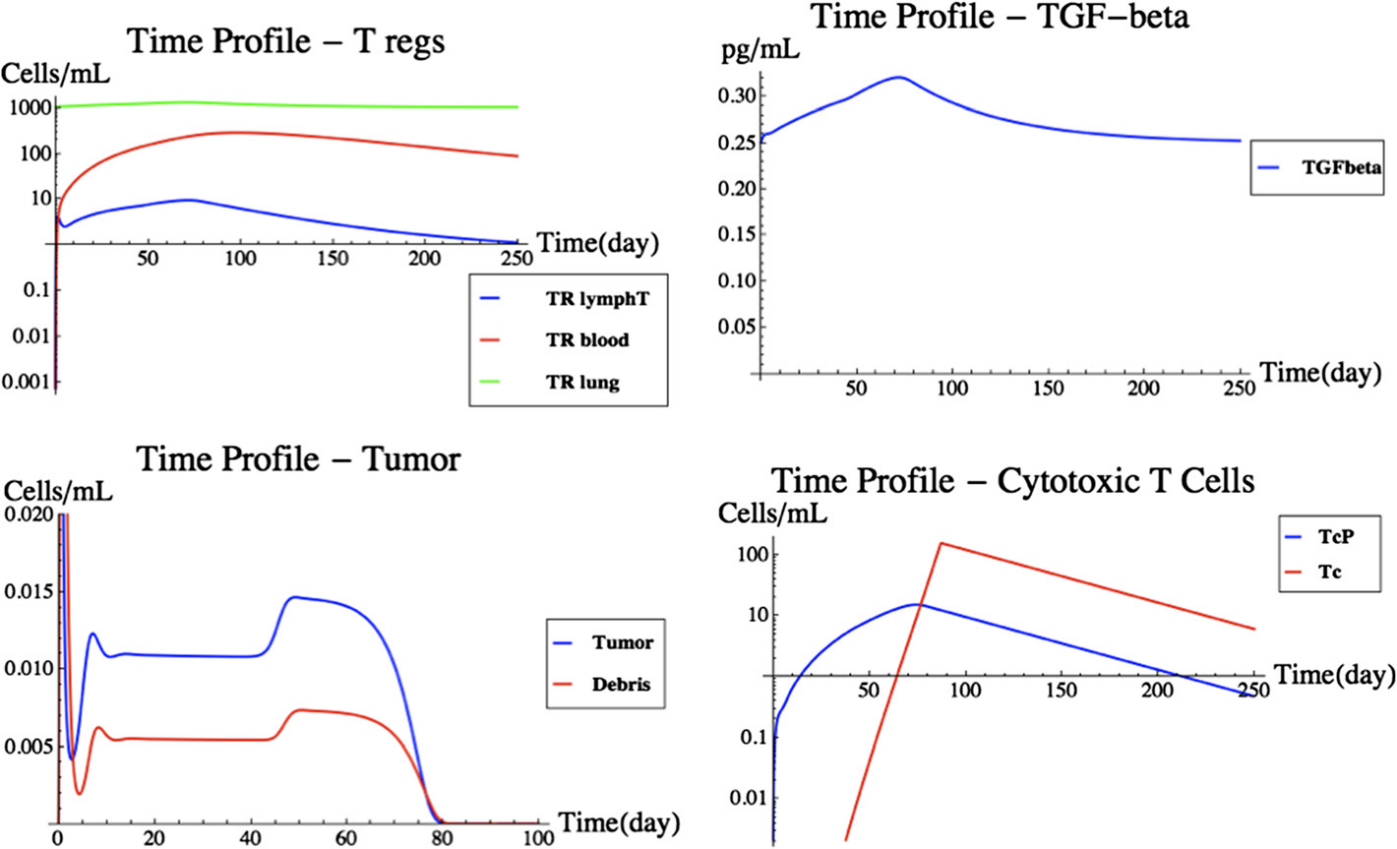
**Tumor removal simulation.** A simulation of spontaneous tumor growth and immune-mediated tumor elimination. See text for details.

FIRM was then simulated with one initial tumor cell in the target organ. This simulation scenario (Figure 9) highlights the multi-scale temporal characteristics of the FIRM simulator. Initially, the tumor grows at a rapid rate, seemingly unchecked. The growth is quickly suppressed by a macrophage response. The activated macrophages reach a level at which the tumor cells are no longer growing, rather settling around a small population size. After this initial response by the macrophages, the presence of the tumor triggers a cellular response. This T-cell response requires a longer time period in order to proliferate to a population size capable of eliminating tumor cells in the target organ. Once the cytotoxic T-cells reach a critical level, the cellular response is able to eliminate the tumor. Once the tumor has been eradicated, the cell populations move back to their steady state levels. This simulation calls into play the features of the DB model [5], with one important difference. While the DB model contained phenomenological features, these have been mostly replaced in the integrated FIRM with more mechanistic cell populations and fluxes which better reflect physiology. It is reassuring that the outcome of the simulation resembles the original model, but it does so with more mechanistic detail about the cellular populations involved. Generic inflammatory signals are still used to represent the kinetics of IL-1 and IL-2. It is worthwhile to mention that tumor secretion of TGF-β would be expected to contribute to these processes by promoting immunoevasion: this is discussed in the next case study, where regulatory T-cells are integrated in the simulation.

What truly happens in the mammalian immune system following challenge with a single proliferating tumor cell is unknown. However, it has been assumed that the immune system regularly removes tumor cells that may arise spontaneously (the concept of immunosurveillance[35]). If anything, this simulation highlights the role played by the T helper population interaction with the tumor. In our hands, a change in this population’s reported half life to 0.02 day^-1^ from 0.3333 day^-1^ was sufficient to produce a more realistic output; however, the model structure may also have been incorrectly specified in the original models. Overall, these considerations point to experimental and research areas of focus. In addition, other parameters in the model may have similar strong influences on the model predictions.

### Influence of T_reg_ on tumor rejection

**Figure 10:**
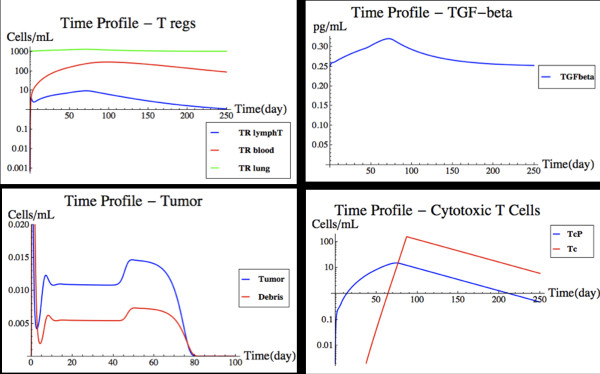
**Tumor removal with regulatory T-cells.** This figure shows a modified version of Figure 7 after T_reg_ and TGF-β have been introduced in the model. from left to right and top to bottom, the time profiles of regulatory T-cells, TGF-β, tumor cells, and cytotoxic T-cells are shown. See text for details.

Regulatory T-cells are resident in tissues and can also be activated through differentiation in lymph nodes through antigen presentation by dendritic cells, with subsequence migration to the site of action. Several cytokines are now known to be involved in this process. The introduction of T_reg_ and TGF-β into the system has a profound effect, as can be seen in Figure 10. The tumor profiles behave similarly as to the previous scenario until about day 45. The tumor experiences growth and is then rejected. The delay in the rejection of the tumor is due to the hampered proliferation of the T-cell population in the target organ, caused by having added T_reg_ and TGF-β dynamics to the model. It essentially takes the T-cell population over 60 days to reach an effective tumor killing level, while previously that took only 45 days.

The T_reg_ addition had a profound effect on how the comprehensive system reacts to the presence of a tumor. As such, it is a good example of how the FIRM simulator can be expanded to include additional, more realistic characteristics of immunological responses. This addition reduces to practice how FIRM could be expanded towards the ultimate goal of building a more comprehensive mathematical representation of immune system features.

## Conclusions

Inflammation and immune response are thought to be a common denominator in human disease. A comprehensive simulator of the immune response in human tissues is thus a needed tool for a variety of applications. FIRM was undertaken as an initial step towards meeting this need. The development and use of FIRM showed that: (1) already developed and proven methods from systems biology could be used to facilitate the construction of a platform for the integration of subset models each focusing on a feature of the immune response; (2) these methods can be successfully implemented to generate an integrated simulator; and (3) that FIRM can account for a variety of challenges to the human immune system through its multi-scale characteristics. A structure such as FIRM can be used prospectively for iterative model building, as the scope of the simulator grows and as new discoveries are made and integrated in the framework; at the same time, the predictions from FIRM can be compared against experimental data, to improve the model and, consequently, mechanistic understanding of its underlying biology.

As molecular systems biology has developed over the past decade, large-scale and even genome-scale models have been formulated [36]. These models are formed from reconstructed networks based on biochemical, genetic and genomic data, and thus have been able to compute a variety of phenotypic functions. This process has been particularly successful for metabolic models [37]. Since such models are mostly based on the universal principles of flux or mass balance, this process can be applied to systems analysis of complex dynamic systems, such as the immune response. We were able to build FIRM based on a reconstructed network of the main cellular and molecular components involved in the immune response. The key challenge over the individual component models is that FIRM is built on multiple tissue spaces of different volumes, even varying volumes in some cases. Additionally, the model equations are formulated in terms of total counts of each variable in a given tissue space. This allows for complete and accurate accounting and balancing of all state variables. Concentrations of cellular and molecular species can then be computed by simply dividing the total amount at each time point with the biological volume of the space, thus allowing for direct comparison to experimental measurements.

The reconstructed network was then populated with information from published models that describe subsets of the immune response. These published models contained detailed information about the mathematical form of the flux equations. In addition, numerical values for all the parameters are provided in these subset models that are typically obtained from measurements or the available experimental literature.

The putative reconstructed network allows the mapping of multiple subset models and their ready integration under a unified format. In principle this is a simple process, but in practice it has been implemented as a stepwise procedure that reveals subtle integration challenges as additional subset cellular and regulatory processes are added. One of the major integration problems arises when discrepancies in the numerical values for the same parameter appear in different subset models. For example, in some cases, assumptions about the physiological processes accounted for in a subset model were not needed in the comprehensive network reconstruction and thus had to be relaxed or otherwise modified (just as an example, eliminating “HTL” (TH1) from macrophage activation in the DB model). This was relatively easy to accomplish since the network reconstruction has a manageable amount of biological and biochemical detail.

Integration of three T-cell responses, the full B-cell response and the regulatory action of key growth factors was performed. All these subsets of the immune response form a postulated, coherent whole as described by FIRM, which by its integrated nature is more comprehensive than any of the individual subsets and can be further expanded for additional realism. FIRM is thus capable of simulating both tumor and pathogen challenges to the immune system, either separately, or simultaneously. When FIRM is applied to simulate such challenges, it displays appealing multi-scale (time, cellular events, etc.) characteristics. This was demonstrated through case studies representing the formation and eradication of a tumor, and the response to TB infection. The challenges encountered in integrating the FIRM network and its component models are actually representative of the obstacles that can be encountered when attempting to synthesize published mathematical models in a cohesive whole. All biological system models are usually delimited by the boundaries of the system being studied – they are not comprehensive because they cannot be. Their scope is usually limited to the original question they were designed to answer. The biological modeling community is currently entering a new stage: it already moved from the development of individual models to the definition of databases and repositories for these models to be shared; the next evolution is to provide robust tools for on-demand component model integration. This task is addressed in this work. When performing model integration, care must be taken not to reach over the scope of the original component models, especially by trying to incorporate or match aspects that cannot be generalized. This is best decided on a case by case basis. FIRM is a starting point for the modeling community to consider these issues.

Given its current state and the iterative model building enabled by the systems biology approach used in its development, FIRM can and should be expanded to account for additional components of the immune response as new knowledge becomes available. An important area where FIRM could be expanded is the innate response, including natural killer (NK) cells. Models have been recently developed [6] to characterize this response component. It would also be useful to examine the behavior of FIRM in the context of reinfection, through refinement of the memory B-cell models and kinetics. In addition, a mechanism for antibody-dependent cellular cytotoxicity (ADCC) would provide an unambiguous link between the DB and the BL model and would enrich the FIRM structure. Perhaps the most important missing feature from FIRM is the activation of the B-cell cascade by the T-helper 2 cells, which is a known, essential mechanism for humoral response initiation. All these remain important areas for further development, whose investigation is made somewhat easier by FIRM’s modular structure.

Since it is possible that a range of parameter values could provide similar unperturbed state values, another area of future investigation would involve sensitivity analysis of the model. To explore this, the model could be expanded to include stochastic behavior so as to account for random variation of the state variables around a physiological set point. Additionally, the model could incorporate expected daily fluctuations, such as e.g. circadian changes, to account for the fact that basal states exhibit a range of behaviors around a baseline. Such analyses might be best performed when the model is used in conjunction with experimental studies, such that development of FIRM (or similar models) can be supported by joint simulations and experimentation.

This work exemplified an approach to constructing large-scale integrated simulators of complex dynamic structures such as the immune system. Ours is a hybrid approach bringing together conventional differential equation-based models with methods developed in systems biology over the past decade. The implementation of this approach leads to a postulated representation of the immune system that incorporates the underlying cellular processes and cytokine regulation based on elements of mammalian physiology. The simulator provided by FIRM is well suited to go through an integrated and interactive model building process with experimental validation to reach an increasing state of completion, similar to what as has been accomplished for genome-scale models of metabolism [37-39].

## Competing interests

TH, EP, MZ, KJ, PO, MES and PV are or were employees of Pfizer.

## Authors’ contributions

SP, TH, EP, MZ, KJ, PO, MES, BOP and PV designed the research; SP, TH, EP, BOP and PV performed the research and SP, TH, EP, MZ, KJ, PO, MES, BOP and PV wrote the manuscript. All authors read and approved the final manuscript.

## Supplementary Material

Additional file 1 **Supplemental Figures and Tables.** FIRM Manuscript Supplemental Figures. Click here for file
