# Supplementary material for: The development of a fully-integrated immune response model (FIRM) simulator of the immune response through integration of multiple subset models
Source: BMC Syst Biol. 2013 Sep 28;7:95. doi: 10.1186/1752-0509-7-95 (PMC3853972; doi:10.1186/1752-0509-7-95)
Supplement: Additional file 1 — Supplemental Figures and Tables. FIRM Manuscript Supplemental Figures. [file 1752-0509-7-95-S1.docx]

# The Development of a Fully-Integrated Immune Response Model (FIRM) Simulator of the Immune Response through Integration of Multiple Subset Models

**Supplemental Figures**

S1 (Figure) – Stoichiometric Matrix

S2 (Table) – Variable Definitions

S3 (Table) – Flux Definitions

S4 (Table) – Rate Laws

S5 (Table) – Kinetic Constants

S6 (Table) – Miscellaneous Rate Law Parameters

S7 (Table) – Compartment Volumes

S8 (Table) – Inactive Flux List

S9 (Figure) – FIRM Re-creation of Figure 2A from the MK Model

S10 (Table) – Sample QC/QA document

**Figure S1 – The stoichiometric matrix that defines FIRM’s network structure**


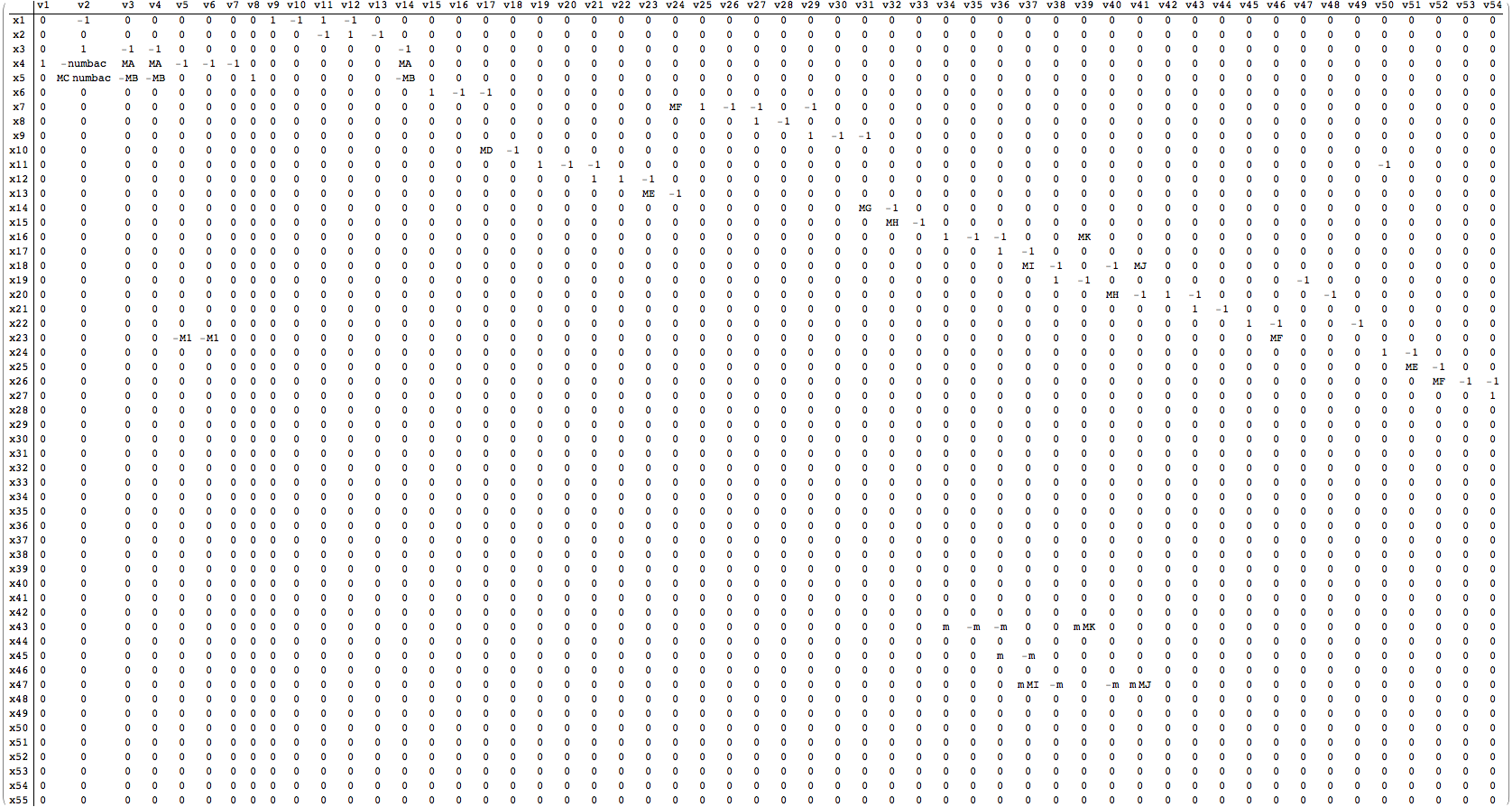


**Figure S1 - (continued)**


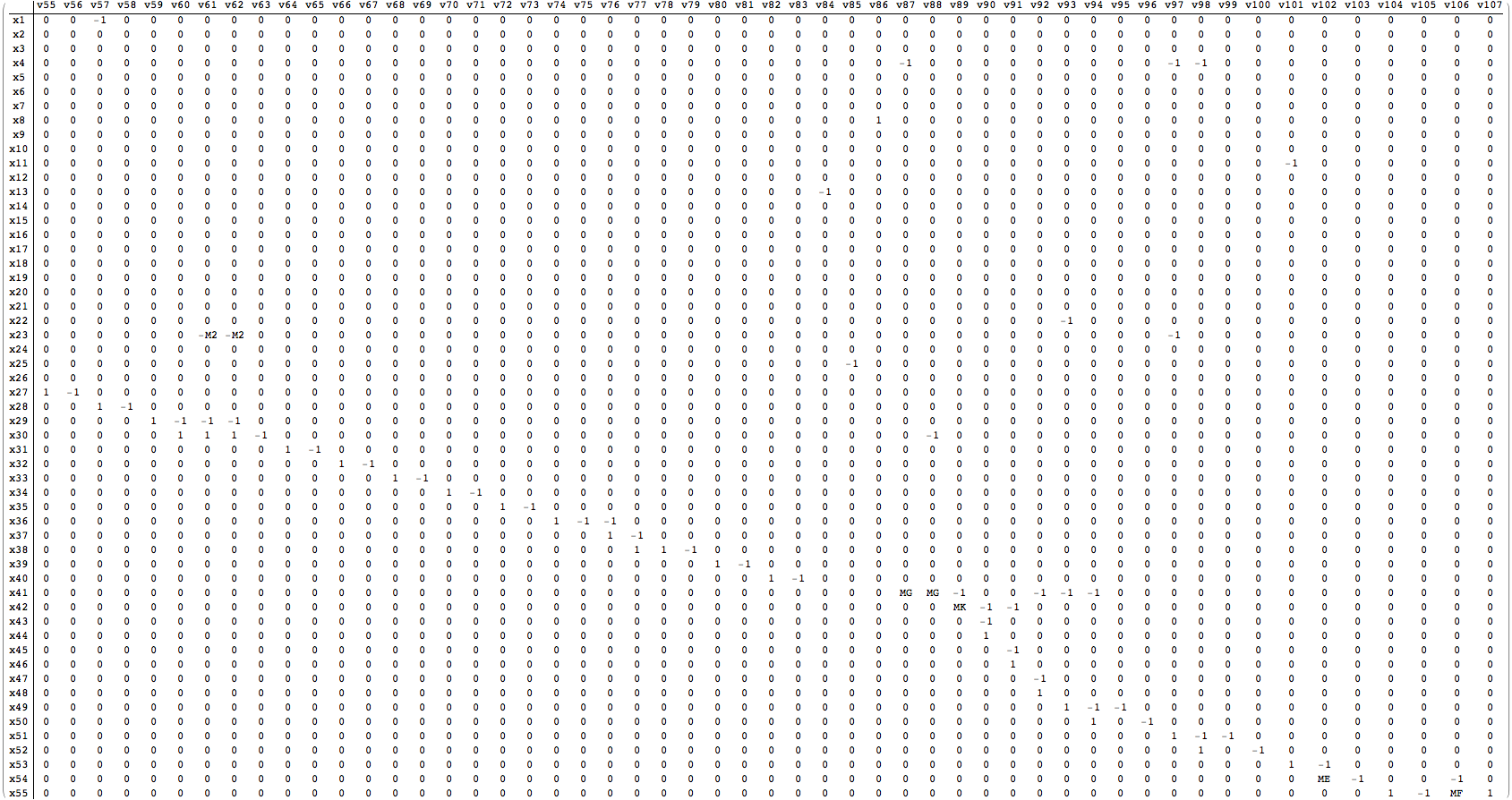


| *Variable* | *Variable Name* | *Variable Definition* |
| --- | --- | --- |
| x1 | M_R_ | Resting macrophage in the target organ |
| x2 | M_A_ | Activated macrophage in the target organ |
| x3 | M_I_ | Infected macrophage in the target organ |
| x4 | P_E_ | Extra-cellular bacteria in the target organ |
| x5 | P_I_ | Intra-cellular bacteria in the target organ |
| x6 | IDC | Immature dendritic cell in the target organ |
| x7 | T_HP_ | T helper precursor cell in the target organ. |
| x8 | T_H1_ | T cell helper type 1 in the target organ. |
| x9 | T_H2_ | T cell helper type 2 in the target organ. |
| x10 | MDC | Mature dendritic cell in the lymphoid T. |
| x11 | T | Naive T cell in the lymphoid T |
| x12 | T_HP_ | T helper precursor cell in the lymphoid T |
| x13 | T_HP_ | T helper precursor cell in the blood. |
| x14 | T_H2_ | T helper 2 cells in the blood |
| x15 | T_H2_ | T helper 2 cells in the lymphoid B |
| x16 | B | Naive B cells at the site of recognition |
| x17 | B_A_ | Activated B cells at the site of recognition |
| x18 | B_A_ | Activated B cells in the blood |
| x19 | B_M_ | Memory B cells in the blood |
| x20 | B_A_ | Activated B cells in the lymphoid B |
| x21 | B_P_ | Plasma B cells in the lymphoid B |
| x22 | AB | Antibodies in the blood |
| x23 | AB | Antibodies in the target organ |
| x24 | T_CP_ | Cytotoxic T cell precursors in the lymphoid T |
| x25 | T_CP_ | Cytotoxic T cell precursors in the blood |
| x26 | T_CP_ | Cytotoxic T cell precursors in the target organ |
| x27 | T_C_ | Cytotoxic T cells in the target organ |
| x28 | M_APC_ | Antigen presenting macrophages in the target organ |
| x29 | TUMOR | Tumor cells in the target organ |
| x30 | DEBRIS | Tumor debris cells in the target organ |
| x31 | I_1_ | Interleukin-1 |
| x32 | I_2_ | Interleukin-2 |
| x33 | I_4_ | Interleukin-4 in the target organ |
| x34 | I_4_ | Interleukin-4 in the lymphoid B |
| x35 | I_10_ | Interleukin-10 |
| x36 | I_12_ | Interleukin-12 in the target organ |
| x37 | I_12_ | Interleukin-12 in the blood |
| x38 | I_12_ | Interleukin-12 in the lymphoid T |
| x39 | Iγ | IFN-gamma |
| x40 | T_β_ | TGF-beta |
| x41 | AG | Antigen in the blood |
| x42 | AG | Antigen in the site of recognition |
| x43 | R | Free receptors sites on naïve B cells |
| x44 | R-AG | Bound receptors sites on naïve B cells |
| x45 | R | Free receptor sites on B_A_ in the site of recognition |
| x46 | R-AG | Bound receptor sites on B_A_ in the site of recognition |
| x47 | R | Free receptor sites on B_A_ in the blood |
| x48 | R-AG | Bound receptor sites on B_A_ in the blood |
| x49 | AB_1_ | Single antigen-bound antibody in the blood |
| x50 | AB_2_ | Double antigen-bound antibody in the blood |
| x51 | AB_1_ | Single antigen-bound antibody in the target organ |
| x52 | AB_2_ | Double antigen-bound antibody in the target organ |
| x53 | T_reg_ | Regulatory T cells in the lymphoid T |
| x54 | T_reg_ | Regulatory T cells in the blood |
| x55 | T_reg_ | Regulatory T cells in the target organ |

**Table S2 – Definition of variables in the integrated model.**

The Variable column indicates the symbol used in the integrated model, the Variable Name column attempts to refer to the original nomenclature of the three models and the Variable Definition column provides a description of each particular state variable.

**Table S3 (start) – Definition of Fluxes in the Integrated Model.**

The Type column attempts a basic classification of fluxes in the model.

| *Flux* | *Flux Description* | *Type* |
| --- | --- | --- |
| v1 | Growth of extra cellular-bacteria in the target organ. | Growth |
| v2 | Infection of a resting macrophage by a extra-cellular bacteria to create an infected macrophage in the target organ. | Binding/Entry |
| v3 | Release of bacteria from intra-cellular to extra-cellular when an infected macrophage bursts in the target organ. | Bursting |
| v4 | Release of bacteria from intra-cellular to extra-cellular when a macrophage kills an infected macrophage. | Apoptosis |
| v5 | Killing of extra-cellular bacteria by an activated macrophage in the target organ. | Apoptosis |
| v6 | Killing of extra-cellular bacteria by an resting macrophage in the target organ. | Apoptosis |
| v7 | Uptake of an extra-cellular bacteria by an immature dendritic cell in the target organ. | Uptake |
| v8 | Growth of intra-cellular bacteria within an infected macrophage in the target organ. | Growth |
| v9 | Recruitment of resting macrophages to the target organ. | Recruitment |
| v10 | Death of resting macrophages in the target organ. | Death - Necrosis |
| v11 | Deactivation of an activated macrophages into a resting macrophages in the target organ. | Differentiation |
| v12 | Activation of a resting macrophage into an activated macrophage in the target organ. | Differentiation |
| v13 | Death of an activated macrophage in the target organ. | Death - Necrosis |
| v14 | Death of an infected macrophage also releasing extra-cellular bacteria into the target organ. | Death - Necrosis |
| v15 | Generation of immature dendritic cells in the target organ. | Recruitment |
| v16 | Death of immature dendritic cells in the target organ. | Death - Necrosis |
| v17 | Migration of immature dendritic cells from the target organ to the lymph node via the afferent lymphatic vessel. | Migration |
| v18 | MDC death in lymphoid T. | Death - Necrosis |
| v19 | Recruitment of naive T cells by MDC in lymphoid T | Recruitment |
| v20 | Death of naive T cells in the lymphoid T | Death - Necrosis |
| v21 | Differentiation of naive T cells to T helper precursor cells in the lymphoid T | Differentiation |
| v22 | T helper cell precursor proliferation in the lymphoid T | Growth |
| v23 | Migration of T helper precursor cells from the lymphoid T to the blood. | Migration |
| v24 | Migration of T helper precursor cells from the blood to the target organ | Migration |
| v25 | Proliferation of T helper precursor cells in the target organ | Growth |
| v26 | Death of T helper precursor cells in the target organ | Death - Necrosis |
| v27 | THP differentiation to T helper type 1 in the target organ | Differentiation |
| v28 | Death of T helper type 1 in the target organ | Death - Necrosis |
| v29 | THP differentiation to T helper type 2 in the target organ | Differentiation |
| v30 | Death of T helper type 2 in the target organ | Death - Necrosis |
| v31 | Migration of T helper 2 cells from the target organ to the blood | Migration |
| v32 | Migration of T helper 2 cells from the blood to the lymphoid B | Migration |
| v33 | Death and circulation of T helper 2 cells in the lymphoid B | Death - Necrosis |
| v34 | Recruitment of naive B cells at the site of recognition | Recruitment |
| v35 | Death of naive B cells at the site of recognition | Death - Necrosis |
| v36 | B cell activation at the site of recognition | Differentiation |
| v37 | Migration of activated B cells from the site of recognition to the blood | Migration |
| v38 | Differentiation of activated B cells to memory B cells in the blood | Differentiation |
| v39 | Migration of memory B cells to the site of recognition and conversion to naive B cells | Migration |
| v40 | Migration of activated B cells from the blood to the lymphoid B | Migration |
| v41 | Migration of activated B cells from the lymphoid B to the blood | Migration |
| v42 | Proliferation of activated B cells in the lymphoid B | Growth |
| v43 | Differentiation of activated B cells to plasma B cells in the lymphoid B | Differentiation |
| v44 | Death of plasma B cells in the lymphoid B | Death - Necrosis |
| v45 | Release (production) of antibodies by plasma b cells in the blood | Secretion |
| v46 | Migration of antibodies from the blood to the target organ | Migration |
| v47 | Slow death of memory B cells in the blood | Death - Necrosis |
| v48 | Death of activated B cells in the lymphoid B | Death - Necrosis |
| v49 | Decay of antibodies in the blood | Death - Decay |
| v50 | Differentiation of naive T cells to cytotoxic T cell precursors in the lymphoid T | Differentiation |
| v51 | Migration of cytotoxic T cell precursors from the lymphoid T to the blood | Migration |
| v52 | Migration of cytotoxic T cell precursors from the blood to the target organ | Migration |
| v53 | Death of cytotoxic T cell precursors in the target organ | Death - Necrosis |
| v54 | Differentiation of cytotoxic T cell precursors to cytotoxic T cells in the target organ | Differentiation |
| v55 | Proliferation of cytotoxic T cells in the target organ | Growth |
| v56 | Death of cytotoxic T cells in the target organ | Death - Necrosis |
| v57 | Conversion of a resting macrophage to an antigen presenting macrophage when in contact with a debris cell in the target organ | Differentiation |
| v58 | Death of antigen presenting macrophages in the target organ | Death - Necrosis |
| v59 | Growth of tumor cells in the target organ | Growth |
| v60 | Natural death of tumor cells in the target organ becoming a debris cell | Death - Necrosis |
| v61 | Killing of tumor cells by an activated macrophage in the target organ | Apoptosis |
| v62 | Killing of tumor cells by a cytotoxic T cell in the target organ | Apoptosis |
| v63 | Decay/clearing of debris cells in the target organ | Death - Removal |
| v64 | Production of Interleukin-1 in the target organ | Production |
| v65 | Decay of Interleukin-1 in the target organ | Death - Decay |
| v66 | Production of Interleukin-2 in the target organ | Production |
| v67 | Decay of Interleukin-2 in the target organ | Death - Decay |
| v68 | Production of Interleukin-4 in the target organ | Production |
| v69 | Decay of Interleukin-4 in the target organ | Death - Decay |
| v70 | Production of Interleukin-4 in the lymphoid B | Production |
| v71 | Decay of Interleukin-4 in the lymphoid B | Death - Decay |
| v72 | Production of Interleukin-10 in the target organ | Production |
| v73 | Decay of Interleukin-10 in the target organ | Death - Decay |
| v74 | Production of Interleukin-12 in the lymphoid T | Production |
| v75 | Decay of Interleukin-12 in the lymphoid T | Death - Decay |
| v76 | Migration of Interleukin-12 from the lymphoid T to the blood | Production |
| v77 | Migration of Interleukin-12 from the blood to the target organ | Death - Decay |
| v78 | Production of Interleukin-12 in the target organ | Production |
| v79 | Decay of Interleukin-12 in the target organ | Death - Decay |
| v80 | Production of IFN-gamma in the target organ | Production |
| v81 | Decay of IFN-gamma in the target organ | Death - Decay |
| v82 | Production of TGF-beta in the target organ | Production |
| v83 | Decay of TGF-beta in the target organ | Death - Decay |
| v84 | Death of T helper precursors in the blood | Death - Necrosis |
| v85 | Death of cytotoxic T precursors in the blood | Death - Necrosis |
| v86 | Proliferation of T helper 1 cells in the target organ | Growth |
| v87 | Permeation of a bacteria (to an antigen) from the target organ to the blood | Permeation |
| v88 | Permeation of a debris (to an antigen) from the target organ to the blood | Permeation |
| v89 | Permeation of an antigen (bacteria or debris) from the blood to the site of recognition | Permeation |
| v90 | Binding of the antigen (bacteria or debris) to a free receptor site on the naive B cells in the site of recognition | Binding |
| v91 | Binding of the antigen (bacteria or debris) to a free receptor site on the activated B cells in the site of recognition | Binding |
| v92 | Binding of the antigen (bacteria or debris) to a free receptor site on the activated B cells in the blood | Binding |
| v93 | Binding of an antigen to a free antibody in the blood | Binding |
| v94 | Binding of an antigen to a single-bound antibody in the blood | Binding |
| v95 | Removal of a single bound antibody in the blood | Death - Removal |
| v96 | Removal of a double bound antibody in the blood | Death - Removal |
| v97 | Binding of an antigen to a free antibody in the target organ | Binding |
| v98 | Binding of an antigen to a single bound antibody in the target organ | Binding |
| v99 | Removal of a single bound antibody in the target organ | Death - Removal |
| v100 | Removal of a double bound antibody in the target organ | Death - Removal |
| v101 | Differentiation from naïve T cells to Treg in the lymphoid T | Differentiation |
| v102 | Migration of Treg from the lymphoid T to the blood | Migration |
| v103 | Death of Treg in the blood | Death - Necrosis |
| v104 | Constant recruitment of Treg in the target organ | Recruitment |
| v105 | Death of Treg in the target organ | Death - Necrosis |
| v106 | Migration of Treg from the blood to the target organ | Migration |
| v107 | The proliferation of Treg in the target organ | Proliferation |

**Table S3 (end) – Definition of Fluxes in the Integrated Model.**

The Type column attempts a basic classification of fluxes in the model.

**Table S4 (start) – Rate laws in the FIRM model.**

| *Flux* | *Rate Law* |
| --- | --- |
| v1 | μ1*(x4/volLung) |
| v2 | k2*(x1/volLung)*x4/(x4 + c2* volLung) |
| v3 | k3*(x3/volLung)*(x5/x3)^2/((x5/x3)^2 + K3^2) |
| v4 | α4*(x3/volLung)*((x8/x3)/((x8/x3) + c4*volLung)) |
| v5 | α5*(x2/volLung)*(x4/volLung) |
| v6 | α6*(x1/volLung)*(x4/volLung) |
| v7 | k7*(x6/volLung)*(x4/volLung) |
| v8 | μ8*(x5/ vMI)*(1 - (x5/x3)^2/((x5/x3)^2 + K3^2)) |
| v9 | ρ9 + μ9*(x2/volLung + w9*x3/volLung) + μi9*INFLAM |
| v10 | η10*(x1/volLung) |
| v11 | δ11*(x2/volLung)*x35/(x35 + cf11*volLung) |
| v12 | δ12*(x1/volLung)*(x4 + x5)/(c12*volLung + x4 + x5)*x39/(x39 + fi12*x33 + cf12*volLung) + δi12*(x1/volLung)*(x29/(ci12 + x29)) |
| v13 | η13*(x2/volLung) |
| v14 | 0 |
| v15 | ρ15 + μ15*x4/(c15*volLung + x4) + (ρ21 + ρ50)*INFLAM |
| v16 | η16*(x6/volLung) |
| v17 | γ17*(x6/volLung)*x4/(c17*volLung + x4) + γ17*(x6/volLung)*1*INFLAM |
| v18 | η18*(x10/volLymphT) |
| v19 | ρ19 + μ19*(x10/volLymphT) |
| v20 | η20*(x11/volLymphT) |
| v21 | ρ21 + δ21*(x11/volLymphT)*(x10/volLymphT) |
| v22 | μ22*(x12/volLymphT)/(c22 + (x12/volLymphT)^2) |
| v23 | γ23*(x12/volLymphT) |
| v24 | γ24*(x13/volBlood)*x2/(c24*volLung + x2) |
| v25 | μ25*(x7/volLung)*x2/(c25*volLung + x2) |
| v26 | η26*(x7/volLung) |
| v27 | δ27*(x7/volLung)*(x38/ volLung)*(x36/(x36 + fi27*x33 + fii27*x35 + cf27*volLung)) + δi27*(x7/volLung)*APC |
| v28 | η28*(x8/volLung) |
| v29 | δ29*(x7/volLung)*x33/(x33 + fi29*x39 + cf29*volLung) |
| v30 | η30*(x9/volLung) |
| v31 | γ31*(x9/volLung) |
| v32 | γ32*(x14/volBlood) |
| v33 | η33*(x15/volLymphB) |
| v34 | ρ34 |
| v35 | η35*(x16/volSite)*(x43/(x43 + x44 + .00001)) |
| v36 | δ36*(x16/volSite)*(x44/(x43 + x44 + .00001)) |
| v37 | γ37*(x17/volSite) |
| v38 | δ38*(x18/volBlood/2)*(x47/(x47 + x48 + .00001)) |
| v39 | η47*(x19/volBlood) |
| v40 | γ40*(x18/volBlood) |
| v41 | 0 (*γ41*(x20/volLymphB)*) |
| v42 | μ42*(x20/volLymphB)*(x48/(x47 + x48 + .00001)) |
| v43 | δ43*(x20/volLymphB/2)*(x47/(x47 + x48 + .00001)) |
| v44 | η44*(x21/volLymphB) |
| v45 | q45a*(x18/volBlood) + q45b*(x20/volBlood) + q45c*(x21/volBlood) |
| v46 | P46*((x22/volBlood) - (x23/volLung)) |
| v47 | η47*(x19/volBlood) |
| v48 | η48*(x20/volLymphB) |
| v49 | η49*(x22/volBlood) |
| v50 | ρ50 + δ50*(x11/volLymphT)*(x10/volLymphT) |
| v51 | γ51*(x24/volLymphT) |
| v52 | γ52*(x25/volBlood)*x2/(c52*volLung + x2) |
| v53 | η53*(x26/volLung) |
| v54 | δ54*(x26/volLung)*(x29/volLung) |
| v55 | μ55*(x27/volLung)*(FACTOR/(cF + FACTOR))*(c55/(c55 + x40)) |
| v56 | η56*(x27/volLung) |
| v57 | 0 |
| v58 | 0 |
| v59 | μ59*(x29/volLung)/(1. + (x29/c59)) |
| v60 | η60*(x29/volLung) |
| v61 | α61*(x2/volLung)*(x29/(c61 + x29)) |
| v62 | α61*(x27/volLung)*(x29/(c61 + x29)) |
| v63 | k63*(x30/volLung) |
| v64 | 0 |
| v65 | 0 |
| v66 | 0 |
| v67 | 0 |
| v68 | q68a*(x7/volLung) + q68b*(x9/volLung) |
| v69 | η69*(x33/volLung) |
| v70 | 0 |
| v71 | 0 |
| v72 | q72a*(x2/volLung)*((c72*volLung)/(x35 + ci72*x39 + c72*volLung)) + q72b*(x8/volLung) + q72c*(x9/volLung) + q72d*(x7/volLung) + q72e*(x3/volLung) |
| v73 | η73*(x35/volLung) |
| v74 | q74*(x10/volLymphT) |
| v75 | η75*(x36/volLymphT) |
| v76 | 0 |
| v77 | 0 |
| v78 | q78a*(x1/volLung) + q78b*(x2/volLung) |
| v79 | η79*(x38/volLung) |
| v80 | ρ80*((x4 + x5)/(c80*volLung + (x4 + x5)))*(x38/(ci80*volLung + x38)) + q80*x8*(x2/(cii80*volLung + x2)) |
| v81 | η81*(x39/volLung) |
| v82 | q82*(x55/volLung) |
| v83 | η83*(x40/volLung) |
| v84 | η84*(x13/volBlood) |
| v85 | η85*(x25/volBlood) |
| v86 | μ86*(x8/volLung)*(FACTOR/(cf86 + FACTOR)) |
| v87 | P87*((x4/volLung) - (x41/volBlood)) |
| v88 | P88*((x30/volLung) - (x41/volBlood)) |
| v89 | P89*((x41/volBlood) - (x42/volSite)) |
| v90 | β90*((x42/volSite)*(x43/volSite) - (x44/volSite)/K90) |
| v91 | β91*((x42/volSite)*(x45/volSite) - (x46/volSite)/K91) |
| v92 | β92*((x41/volSite)*(x47/volSite) - (x48/volSite)/K92) |
| v93 | β93*((x41/volBlood)*(x22/volBlood) - (x49/volBlood)/K93) |
| v94 | β94*((x41/volBlood)*(x49/volBlood) - (x50/volBlood)/K94) |
| v95 | η95*(x49/volBlood) |
| v96 | η96*(x50/volBlood) |
| v97 | β97*((x4/volLung)*(x23/volLung) - (x51/volLung)/K97) |
| v98 | β98*((x4/volLung)*(x51/volLung) - (x52/volLung)/K98) |
| v99 | η99*(x51/volLung) |
| v100 | η100*(x52/volLung) |
| v101 | w101* ρ101 + w101* δ101*(x11/volLymphT)*(x10/volLymphT) |
| v102 | γ102*(x53/volLymphT) |
| v103 | η103*(x54/volBlood) |
| v104 | ρ104 |
| v105 | η105*(x55/volLung) |
| v106 | γ106*(x54/volBlood)*x2/(c106*volLung + x2) |
| v107 | μ107*(x55/volLung)*(x29/(x29 + c107)) |

**Table S4 (end) – Rate laws in the FIRM model.**

**Table S5 – Numerical values for the kinetic constants (start).**

Abbreviations: MK = Marino-Kirschner (Ref. 10); DB = de Boer (Ref. 5); BL = Bell (Ref. 8).

| *Variable* | *Value* | *Source* | *Description* |
| --- | --- | --- | --- |
| μ1 | 0.005 day^-1^ | MK | Growth rate of extracellular bacteria |
| k2 | 0.4 day^-1^ | MK | Maximum rate of resting macrophage chronic infection |
| k3 | 0.1 day-^1^ | MK | Maximum infected macrophage death rate due to bacteria |
| α4 | 0.5 day^-1^ | MK | Maximum T cell killing of infected macrophages |
| α5 | 1.25*10-7(ml/M_A_) day | MK | Maximum killing of extracellular bacteria by resting macrophages |
| α6 | 1.25*10-8(ml/M_R_) day | MK | Maximum killing of extracellular bacteria by activated macrophages |
| k7 | 10-7 (day IDC)^-1^ | MK | Bacteria uptake rate by IDCs |
| μ8 | 0.1 day^-1^ | MK | Growth rate of intracellular bacteria |
| ρ9 | 5000 M_R_/ml/day | MK | Resting macrophage recruitment |
| μ9 | 0.04 day^-1^ | MK | Resting macrophage recruitment induced by infected and activated macrophages |
| μi9 | 125,000 M_R_/ml /day | DB | Resting macrophage recruitment induced by tumor inflammation reaction |
| η10 | 0.01 day^-1^ | MK | Half-life of resting macrophages |
| δ11 | 0.36 day^-1^ | MK | Maximum rate of macrophage deactivation |
| δ12 | 0.4 day^-1^ | MK | Maximum rate of macrophage activation induced by bacteria |
| δi12 | 0.009 day^-1^ | DB | Activation rate of macrophages induced by tumor cells |
| η13 | 0.01 day^-1^ | MK | Half-life of activated macrophages |
| η14 | 0.01 day^-1^ | MK | Half-life of infected macrophages |
| ρ15 | 500 IDC/ml/day | MK | Baseline IDC in the lung |
| μ15 | 0.02 day^-1^ | MK | Maximum rate of IDC recruitment to the site of infection (due to extracellular bacteria) |
| η16 | 0.01 day^-1^ | MK | Half-life of IDC |
| γ17 | 0.2 day^-1^ | MK | Maximum rate of IDC activation/migration/maturation |
| η18 | 0.02 day^-1^ | MK | Half-life of MDC |
| ρ19 | 1000 (T/ml)/day | MK | Baseline of naïve T cells circulating through the lymph nodes |
| μ19 | 0.1 (T/MDC)/day | MK | Maximum rate of recruitment of naïve T cells in the lymph nodes due to MDC |
| η20 | 0.01 day^-1^ + 0.002 day^-1^ | MK | Half-life of naïve T cells and recirculation of naïve T cells through the lymph nodes |
| δ21 | 0.0001 (day MDC)^-1^ | MK | Maximum naïve T cell activation to helper T precursors by the MDC |
| ρ21 | 100 T_HP_/ml/day | Analogous to DB (I2) | Baseline differentiation of naïve T cells to helper T precursors |
| μ22 | 0.9 day^-1^ | MK | Half-sat of helper T precursors in the lymph nodes |
| γ23 | 0.9 day^-1^ | MK | % of helper T precursors migrating from the lymph nodes to the lung |
| γ24 | 0.9 day^-1^ | MK | % of helper T precursors migrating from the lymph nodes to the lung |
| μ25 | 0.4 day^-1^ | MK | Maximum rate of helper T precursor proliferation induced by activated macrophages |
| η26 | 0.3333 day^-1^ | MK | Half-life of helper T precursors |
| δ27 | 0.1 (ml/pg) day | MK | Rate of Th1 differentiation induced by cytokines |
| δi27 | 0.001 day^-1^ | DB | Th1 activation rate induced by tumor cells |
| η28 | 0.3333 day^-1^ | MK | Half-life of Th1 |
| δ29 | 0.05 day^-1^ | MK | Rate of Th2 differentiation |
| η30 | 0.3333 day^-1^ | MK | Half-life of Th2 |
| γ31 | 0.9 day^-1^ | Similar to γ24 | Migration rate of Th2 from the lung to the blood |
| γ32 | 0.9 day^-1^ | Similar to γ24 | Migration rate of Th2 from the blood to the spleen |
| η33 | 0.3333 day^-1^ | Similar to η30 | Half-life of Th2 |
| ρ34 | 10 B/ml/day | Estimation, Value given to source1(t) in BL | Base line of naïve B cells |
| η35 | 2.4 day^-1^ | BL | Half-life of naïve B cells |
| δ36 | 2.4 day^-1^ | BL | Mean time for naïve B cell differentiation to activated B cells induced by antigen stimulation |
| γ37 | 0.9 day^-1^ | Similar to γ24 | Migration rate of activated B cells from the site of recognition to the blood |
| δ38 | 2.4 day^-1^ | BL | Mean time for activated B cell differentiation to memory B cells induced by antigen stimulation |
| δ39 | 0.0024 day^-1^ | Similar to η47 | Rate of memory B cells migrating and rejoining the naïve B cell population |
| γ40 | 0.9 day^-1^ | Similar to γ24 | Migration rate of activated B cells from the blood to the spleen |
| γ41 | 0.9 day^-1^ | Similar to γ24 | Migration rate of activated B cells from the spleen to the blood |
| μ42 | 2.4 day^-1^ | BL | Mean time for activated B cells to divide induced by antigen stimulation |
| δ43 | 2.4 day^-1^ | BL | Mean time for activated B cell differentiation to plasma B cells induced by antigen stimulation |
| η44 | 1.2 day^-1^ | BL | Half-life of plasma B cells |
| q45a | 1,000,000 (AB/B) day^-1^ | BL | Antibody production by activated B cells in the blood |
| q45b | 1,000,000 (AB/B_A_) day^-1^ | BL | Antibody production by activated B cells in the spleen |
| q45c | 1,000,000 (AB/B_A_) day^-1^ | BL | Antibody production by plasma B cells in the spleen |
| P46 | 9 day^-1^ | Estimation, No Literature Source | Permeation of antibodies between the blood and the target organ |
| η47 | 0.0024 day^-1^ | BL | Half-life of memory B cells |
| η48 | 0.024 day^-1^ | BL | Half-life of activated B cells |
| η49 | 0.048 day^-1^ | BL | Half-life of free antibodies |
| δ50 | 0.0001 (day MDC)^-1^ | Similar to ρ21 | Maximum naïve T cell activation to cytotoxic T precursors by the MDC |
| ρ50 | 100 T_CP_/ml/day | Analogous to DB (I1) | Baseline differentiation of naïve T cells to cytotoxic T precursors |
| γ51 | 0.9 day^-1^ | Similar to γ24 | Migration rate of cytotoxic T cells from the lymph nodes to the blood |
| γ52 | 0.9 day^-1^ | Similar to γ24 | Migration rate of cytotoxic T cells from the blood to the target organ/lung |
| η53 | 0.02 day^-1^ | DB | Half-life of T lymphocytes |
| δ54 | 0.001 (TUMOR day)^-1^ | DB | Differentiation rate of cytotoxic T precursors to cytotoxic T cells |
| μ55 | 1 day^-1^ | DB | Proliferation rate of cytotoxic cells induced by FACTOR |
| η56 | 0.02 day^-1^ | DB | Half-life of T lymphocytes |
| μ59 | 1 day | DB | Growth rate of tumor cells |
| η60 | 0.0001 day^-1^ | DB | Half-life of tumor cells |
| α61 | 10 (M_A_ day)^-1^ | DB | Maximum killing capacity of activated macrophages |
| α62 | 10 (T_C_ day)^-1^ | DB | Maximum killing capacity of cytotoxic T cells |
| η63 | 2 day^-1^ | DB | Half-life of tumor debris |
| q68a | 0.0029 (pg/T_HP_) day^-1^ | MK | IL-4 production by helper T precursors |
| q68b | 0.0218 (pg/T_H2_) day^-1^ | MK | IL-4 production by Th2 |
| η69 | 2.77 day^-1^ | MK | Half-life of IL-4 |
| q72a | 0.006 (pg/M_A_) day^-1^ | MK | Maximum rate of IL-10 production by activated macrophages |
| q72b | 0.00005 (pg/T_H1_) day^-1^ | MK | IL-10 production by Th1 |
| q72c | 0.0001 (pg/T_H2_) day^-1^ | MK | IL-10 production by Th2 |
| q72d | 0.0001 (pg/T_HP_) day^-1^ | MK | Maximum rate of IL-10 production by helper T precursors induced by IL-12 |
| q72e | 0.0001 (pg/M_I_) day^-1^ | MK | IL-10 production by infected macrophages |
| η73 | 3.6968 day^-1^ | MK | Half-life of IL-10 |
| q74 | 0.0035 (pg/MDC) day^-1^ | MK | IL-12 production by MDC |
| η75 | 1.188 day^-1^ | MK | Half-life of IL-12 |
| q78a | 0.00000275 (pg/M_R_) day^-1^ | MK | IL-12 production by activated macrophages |
| q78b | 0.0008 (pg/M_A_) day^-1^ | MK | IL-12 production by resting macrophages |
| η79 | 1 day^-1^ | MK | Half-life of IL-12 |
| ρ80 | 700 pg/ml/day | MK | IFN-gamma production induced by bacteria and IL-12 (other sources) |
| q80 | 0.02 (pg/T_H1_) day^-1^ | MK | IFN-gamma production by Th1 |
| η81 | 3 day^-1^ | MK | Half-life of IFN-gamma |
| q82 | 5 *10‐4 day^‐1^ | Estimation, similar to other cytokines | TGF-beta production by Tregs in the target organ |
| η83 | 2 day^‐1^ | Estimation, similar to other cytokines | Half-life of TGF-beta |
| η84 | 0.3333 day^-1^ | Similar to η26 | Half-life of T lymphocytes |
| η85 | 0.02 day^-1^ | Similar to η53 | Half-life of T lymphocytes |
| μ86 | 1 day^-1^ | DB | Proliferation rate of Th1 induced by FACTOR |
| P87 | 3 day^-1^ | Estimation, No Literature Source | Permeation of bacteria between the target organ and the blood |
| P88 | 3 day^-1^ | Estimation, No Literature Source | Permeation of tumor debris and the blood |
| P89 | 3 day^-1^ | Estimation, No Literature Source | Permeation of the antigen between the blood and the site of recognition |
| β90 | 1000 day^-1^ | Estimation, No Literature Source | Binding rate of an antigen to a receptor site on a naïve B cell |
| β91 | 1000 day^-1^ | Estimation, No Literature Source | Binding rate of an antigen to a receptor site on an activated B cell |
| β92 | 1000 day^-1^ | Estimation, No Literature Source | Binding rate of an antigen to a receptor site on an activated B cell |
| β93 | 1000 day^-1^ | Estimation, No Literature Source | Binding rate of an antigen to an antibody |
| β94 | 1000 day^-1^ | Estimation, No Literature Source | Binding rate of an antigen to an antibody |
| η95 | 4.8 day^-1^ | BL | Mean time for the removal of an antigen bound to an antibody |
| η96 | 4.8 day^-1^ | BL | Mean time for the removal of an antigen bound to an antibody |
| β97 | 1000 day^-1^ | Estimation, No Literature Source | Binding rate of an antigen to an antibody |
| β98 | 1000 day^-1^ | Estimation, No Literature Source | Binding rate of an antigen to an antibody |
| η99 | 4.8 day^-1^ | BL | Mean time for the removal of an antigen bound to an antibody |
| η100 | 4.8 day^-1^ | BL | Mean time for the removal of an antigen bound to an antibody |
| ρ101 | 20 T_reg_/ml/day | Estimation, ρ21+ρ50 | Baseline differentiation of naïve T cells to regulatory T cells |
| δ101 | 0.00002 (MDC day)^‐1^ | Estimation, δ21+δ50 | Maximum naïve T cell activation to regulatory T cells by the MDC |
| γ102 | 0.9 day^‐1^ | Similar to γ24 | Migration of regulatory T cells from the lymphoid T to the blood |
| η103 | 0.02 day^‐1^ | Similar to η53 | Half-life of T lymphocytes |
| ρ104 | 0.00002 Treg /day | Estimation, ρ21+ρ50 | Baseline recruitment of regulatory T cells in the target organ |
| η105 | 0.02 day^‐1^ | Similar to η53 | Half-life of T lymphocytes |
| γ106 | 0.9 day^‐1^ | Similar to γ24 | Migration of regulatory T cells from the blood to the target organ |
| μ107 | 0.5 day^‐1^ | Estimation, No Literature Source | Proliferation rate of regulatory T cells induced by TUMOR cells |

**Table S5 – Numerical values for the kinetic constants (end)**

**Table S6 (start) – Numerical values for the rate parameters**

| *Parameter* | *Value* | *Source* | *Description* | |
| --- | --- | --- | --- | --- |
| c2 | 10^6^ P_E_/ml | MK | Half-maximal saturation of resting macrophages infection induced by extracellular bacteria | |
| K3 | 50 B_I_/M_I_ | MK | Max MOI of infected macrophages | |
| c4 | 0.15 T_H1_/M_I_ | MK | Half-maximal saturation, Th1 to infected macrophage ratio for infected macrophage lysis | |
| w9 | 0.14 | MK | Weight (Scalar) | |
| cf11 | 100 pg/ml | MK | Half-maximal saturation, IL-10 on activated macrophage deactivation | |
| c12 | 5*10^5^ P_total_/ml | MK | Half-maximal saturation, total bacteria on resting macrophage activation | |
| ci12 | 1000 cells | DB | Restimulation saturation | |
| fi12 | 2.333 | MK | Adjustment, IFN-gamma/IL-4 on activated macrophage (Scalar) | |
| cf12 | 150 pg/ml | MK | Half-maximal saturation, IFN-gamma on resting macrophage activation | |
| c15 | 1.5*10^3^ P_E_/ml | MK | Half-maximal saturation, extracellular bacteria on IDC recruitment | |
| c17 | 10^4^ P_E_/ml | MK | Half-maximal saturation, extracellular bacteria on IDC activation/migration/maturation | |
| c22 | 3*10^3^ T/day | MK | Threshold in helper T precursor proliferation | |
| c24 | 1.5*10^4^ M_A_/ml | MK | Half-maximal saturation, activated macrophages on helper T precursor migration | |
| c25 | 10^5^ M_A_/ml | MK | Half-maximal saturation, activated macrophage on helper T precursor proliferation | |
| fi27 | 4.1 | MK | Adjustment, IFN-gamma + IL-12LN / IL-4 on Th1 differentiation (Scalar) | |
| fii27 | 4.8 | MK | Adjustment, IFN-gamma + IL-12LN / IL-10 on Th1 differentiation (Scalar) | |
| cf27 | 30 pg/ml | MK | Half-maximal saturation, IFN-gamma + IL-12LN on Th1 differentiation | |
| fi29 | 0.12 | MK | Adjustment, IL-4/IFN-gamma on Th2 differentiation (Scalar) | |
| cf29 | 2 pg/ml | MK | Half-maximal saturation, IL-4 on Th2 differentiation | |
| c52 | 1.5*10^4^ M_A_/ml | Similar to c24 | Half-maximal saturation, activated macrophages on cytotoxic T precursor migration | |
| c55 | 100 units | DB | Factor saturation | |
| c59 | 10^9^ cells | DB | Growth rate saturation | |
| c61 | 10^5^ cells | DB | Killing saturation | |
| c62 | 10^5^ cells | DB | Killing saturation | |
| c72 | 51 pg/ml | MK | Half-maximal saturation, IL-10 and IFN-gamma on IL-10 | |
| ci72 | 0.05 | MK | Adjustment, IFN-gamma / IL-10 on activated macrophage production of IL-10 (Scalar) | |
| c80 | 5*10^3^ P_total_/ml | MK | Half-maximal saturation, total bacteria on extra IFN-gamma production | |
| ci80 | 50 pg/ml | MK | Half-maximal saturation, IL-12 on extra IFN-gamma production | |
| cii80 | 10^5^ M_A_/ml | MK | Half-maximal saturation, activated macrophage on IFN-gamma production of Th1 | |
| cf86 | 50 units | DB | Factor saturation | |
| K90 | 1,000 (molecules/ml)^-1^ | BL | Binding affinity of the antigen to the receptor site | |
| K90 | 1,000 (molecules/ml)^-1^ | BL | Binding affinity of the antigen to the receptor site | |
| K92 | 1,000 (molecules/ml)^-1^ | BL | Binding affinity of the antigen to the receptor site | |
| K93 | 1,000 (molecules/ml)^-1^ | BL | Binding affinity of the antigen to an antibody | |
| K94 | 1,000 (molecules/ml)^-1^ | BL | Binding affinity of the antigen to an antibody | |
| K97 | 1,000 (molecules/ml)^-1^ | BL | Binding affinity of the antigen to an antibody | |
| K98 | 1,000 (molecules/ml)^-1^ | BL | Binding affinity of the antigen to an antibody | |
| cF | 1000 cells | DB | Restimulation saturation | |
| μI | 9 | DB | Inflammation constant | |
| cI | 50 units | DB | Factor saturation | |
| cAPC | 10^7^ units | DB | Presentation saturation | |
| w101 | 0.1 | Ref. [30] | Weight (Scalar) | |
| c106 | 1.5*10^4^ MA/ml | Similar to c24 | Half-maximal saturation, activated macrophages on regulatory T cell migration | |
| c107 | 1000 cells | Estimation | Half-maximal saturation, TUMOR cells on regulatory T cell proliferation | |
| *Parameter* | ***Value*** | | ***Source*** | ***Description*** |
| FACTOR | x8*(x29/(cF+x29)) | | DB | Lymphoid factors |
| INFLAM | μI*(x29/(cF+x29))/(cI+(x29/cF+x29))) | | DB | Inflammation reaction |
| APC | ((x1+x2)*x30)/(cAPC+x30) | | DB | Antigen presentation |

**Table S6 (end) – Numerical values for the rate parameters**

| *Compartment* | *Volume* |
| --- | --- |
| Lung | 1000 ml |
| Blood | 4500 ml |
| Lymphoid T | 10 ml |
| Lymphoid B | 150 ml |
| Site of Recognition | 4500 ml |
| Infected Macrophage | 16*10^-9^ ml/M_I_ |

**Table S7 – Compartment Volumes in the FIRM model.**

| *Flux* | *Reason for Deactivation* |
| --- | --- |
| v_14_ | This flux had the same functionality as v_3_, therefore the fluxes were combined into one flux (v_3­_). |
| v_30_ | With fluxes v_30_ and v_31_ active, the T_H2_ population was depleted at too high of a rate. To remedy this, both fluxes were combined into one flux (v_31_). |
| v_57_ | The function of M_APC_ (macrophages functioning as antigen presenting cells) is incorporated in the dendritic cell population and not explicitly accounted for. |
| v_58_ | The function of M_APC_ (macrophages functioning as antigen presenting cells) is incorporated in the dendritic cell population and not explicitly accounted for. |
| v_64_ | The functionality of IL-1 is not yet defined in FIRM. |
| v_65_ | The functionality of IL-1 is not yet defined in FIRM. |
| v_66_ | The functionality of IL-2 is not yet defined in FIRM. |
| v_67_ | The functionality of IL-2 is not yet defined in FIRM. |
| v_70_ | The function of T_H2_ in the humoral response was not included due to lack of quantitative data. |
| v_71_ | The function of T_H2_ in the humoral response was not included due to lack of quantitative data. |
| v_76_ | The function of IL-12 in the lymphoid T defined in the MK model did not require presence in the target organ. |
| v_77_ | The function of IL-12 in the lymphoid T defined in the MK model did not require presence in the target organ. |
| v_88_ | The tumor-antibody interaction was not included due to lack of quantitative data. Therefore, tumor debris will not migrate into the blood. |

**Table S8 – List of inactive fluxes in the final FIRM structure.**

While IL-1 is not explicitly represented in the mass action model, its functionality is summarized in the parameter INFLAM.

| 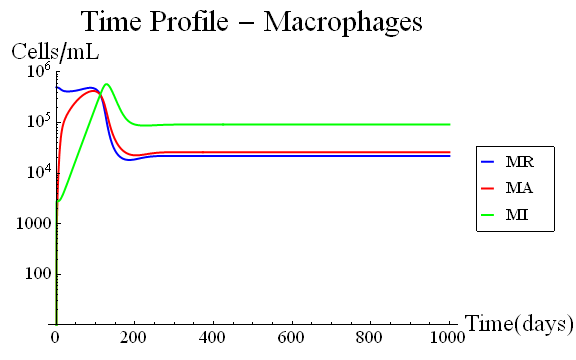 | 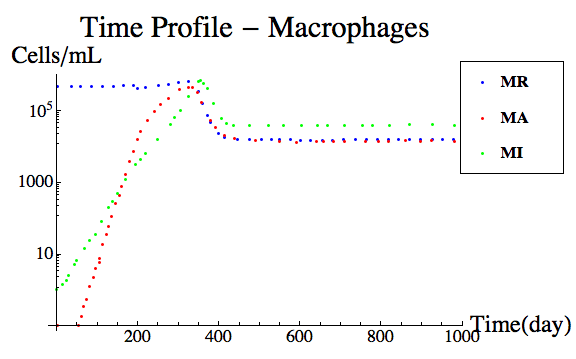 |
| --- | --- |
| 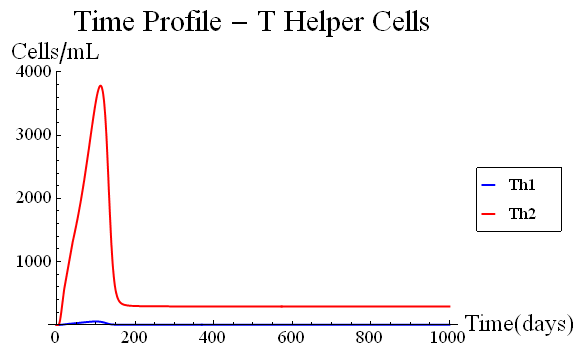 | 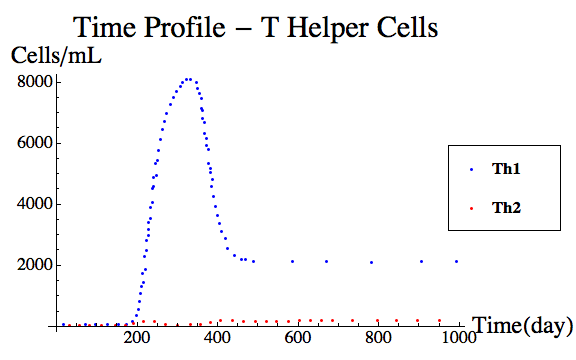 |
| 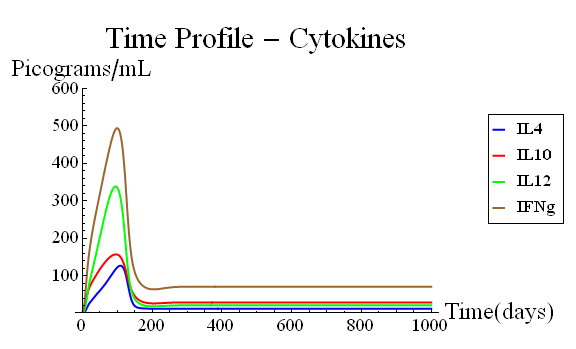 |  |
| 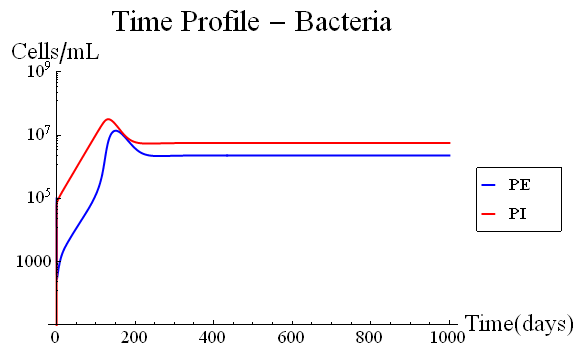 | 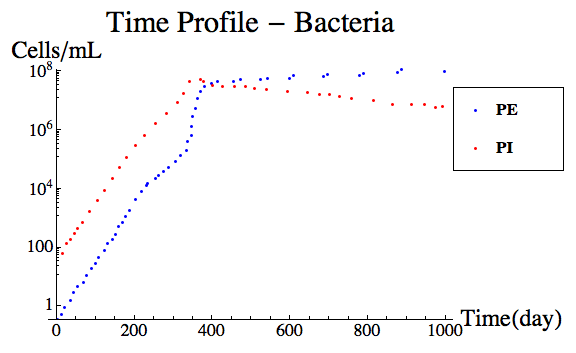 |

Figure S9 – A FIRM re-creation (left) of Figure 2A from the published MK model (right) original reference [Ref. 10] on TB infection in the lung.

This simulation was performed at nominal parameter values. There are a few differences in the two figures, but this is expected given that there were changes made when mapping the original model onto the FIRM structure.

# Figure S10 - Sample QC/QA Check: Bacteria Accounting

QC/QA Analysis of the FIRM Model’s Accounting of Intra/Extracellular Bacteria

The FIRM model includes a description of infection of resting macrophages and the release of bacteria when an infected macrophage bursts or is eliminated. As a numerical check, we can turn off the growth and clearance rates of bacteria (v_1_, v_5_, v_6_, v_7_, v_8_ = 0) to demonstrate quantitative and accurate accounting of bacteria in the system. The flux associated with infection is v_2_ and the flux associated with release is v_3_. There are three scenarios that will be tested:

1. Only flux v_2_ is active
2. Only flux v_3_ is active
3. Both fluxes v_2_ and v_3_ are active

Resting macrophage recruitment (v_9_) and death (v_10_) are active for these tests, as they are necessary for infection and a complete infection/bursting cycle.

**Scenario 1**. The flux vector in the Mathematica notebook is:

fluxes = {0, v2, 0, 0, 0, 0, 0, 0, v9, v10, 0, 0, 0, 0, 0, 0, 0, 0, 0, 0, 0, 0, 0, 0, 0, 0, 0, 0, 0, 0, 0, 0, 0, 0, 0, 0, 0, 0, 0, 0, 0, 0, 0, 0, 0, 0, 0, 0, 0, 0, 0, 0, 0, 0, 0, 0, 0, 0, 0, 0, 0, 0, 0, 0, 0, 0, 0, 0, 0, 0, 0, 0, 0, 0, 0, 0, 0, 0, 0, 0, 0, 0, 0, 0, 0, 0, 0, 0, 0, 0, 0, 0, 0, 0, 0, 0, 0, 0, 0, 0}

The initial conditions are set to:

varstst = {500000000, 0, .0000001, 1000, 0, 0, 0, 0, 0, 0, 0, 0, 0, 0, 0, 0, 0, 0, 0, 0, 0, 0, 0, 0, 0, 0, 0, 0, 0, 0, 0, 0, 0, 0, 0, 0, 0, 0, 0, 0, 0, 0, 0, 0, 0, 0, 0, 0, 0, 0, 0, 0}

Graphing the populations (Figure 1) of intracellular bacteria (x_5_) and extracellular bacteria (x_4_) against time shows that they mirror each other during infection. This means that as one extracellular bacterium infects a resting macrophage, one intracellular bacterium appears.

Graphing the total bacteria population (x_4_ + x_5_) produces a straight line. This shows the bacteria population is preserved in the system when bacteria killing, uptake, and growth fluxes are inactive.


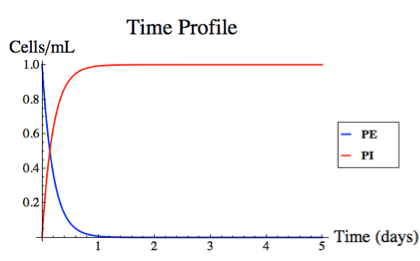


Figure 1 – Bacteria accounting with only flux v_2_ active and an initial bacteria load of 1000 in the extracellular compartment. Results show that bacteria in the system are preserved.

**Scenario 2**. The flux vector in the Mathematica notebook is:

fluxes = {0, 0, v3, 0, 0, 0, 0, 0, v9, v10, 0, 0, 0, 0, 0, 0, 0, 0, 0, 0, 0, 0, 0, 0, 0, 0, 0, 0, 0, 0, 0, 0, 0, 0, 0, 0, 0, 0, 0, 0, 0, 0, 0, 0, 0, 0, 0, 0, 0, 0, 0, 0, 0, 0, 0, 0, 0, 0, 0, 0, 0, 0, 0, 0, 0, 0, 0, 0, 0, 0, 0, 0, 0, 0, 0, 0, 0, 0, 0, 0, 0, 0, 0, 0, 0, 0, 0, 0, 0, 0, 0, 0, 0, 0, 0, 0, 0, 0, 0, 0}

The initial conditions are set to:

varstst = {500000000, 0, .0000001, 0, 1000, 0, 0, 0, 0, 0, 0, 0, 0, 0, 0, 0, 0, 0, 0, 0, 0, 0, 0, 0, 0, 0, 0, 0, 0, 0, 0, 0, 0, 0, 0, 0, 0, 0, 0, 0, 0, 0, 0, 0, 0, 0, 0, 0, 0, 0, 0, 0}

Graphing the populations (Figure 2) of intracellular bacteria (x_5_) and extracellular bacteria (x_4_) against time shows that they mirror each other when bursting occurs. This means that as one intracellular bacterium is released by a bursting infected macrophage, one extracellular bacterium appears.


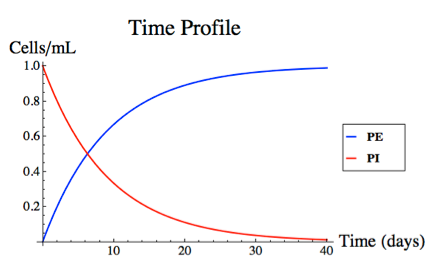

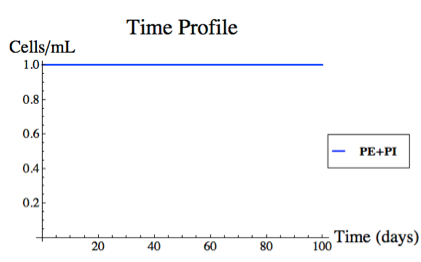


Figure 2 – Bacteria accounting with only flux v_3_ active and an initial bacteria load of 1000 in the intracellular compartment. Results show that bacteria in the system are preserved.

Graphing the total bacteria population (x_4_ + x_5_) produces a straight line. This shows the bacteria population is kept constant in the system when bacteria killing, uptake, and growth fluxes are inactive.

**Scenario 3**. The flux vector in the Mathematica notebook is:

fluxes = {0, v2, v3, 0, 0, 0, 0, 0, v9, v10, 0, 0, 0, 0, 0, 0, 0, 0, 0, 0, 0, 0, 0, 0, 0, 0, 0, 0, 0, 0, 0, 0, 0, 0, 0, 0, 0, 0, 0, 0, 0, 0, 0, 0, 0, 0, 0, 0, 0, 0, 0, 0, 0, 0, 0, 0, 0, 0, 0, 0, 0, 0, 0, 0, 0, 0, 0, 0, 0, 0, 0, 0, 0, 0, 0, 0, 0, 0, 0, 0, 0, 0, 0, 0, 0, 0, 0, 0, 0, 0, 0, 0, 0, 0, 0, 0, 0, 0, 0, 0}

The initial conditions are set to:

varstst = {500000000, 0, .0000001, 0, 1000, 0, 0, 0, 0, 0, 0, 0, 0, 0, 0, 0, 0, 0, 0, 0, 0, 0, 0, 0, 0, 0, 0, 0, 0, 0, 0, 0, 0, 0, 0, 0, 0, 0, 0, 0, 0, 0, 0, 0, 0, 0, 0, 0, 0, 0, 0, 0}

Graphing the populations (Figure 3) of intracellular bacteria (x_5_) and extracellular bacteria (x_4_) against time shows that they mirror each other during a complete infection/bursting cycle. Since the infection rate (v_2_) is much faster than the bursting rate, the majority of the total bacteria population will be intracellular bacteria.

Graphing the total bacteria population (x_4_ + x_5_) produces a straight line. This shows the bacteria population is preserved in the system when bacteria killing, uptake, and growth fluxes are inactive.


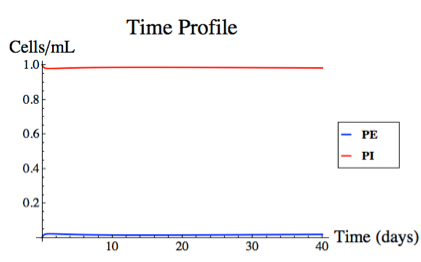

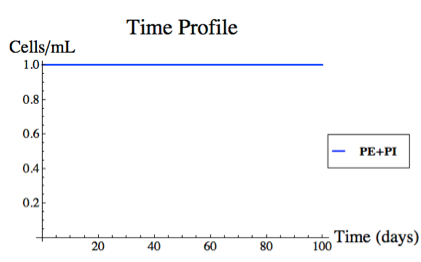


Figure 3 – Bacteria accounting with both fluxes v_2_ and v_3_ active, and an initial bacteria load of 1000 in the intracellular compartment. Results show that bacteria in the system are preserved.
